# Role of the Discriminator Sequence in the Supercoiling Sensitivity of Bacterial Promoters

**DOI:** 10.1128/mSystems.00978-21

**Published:** 2021-08-24

**Authors:** Raphaël Forquet, Maïwenn Pineau, William Nasser, Sylvie Reverchon, Sam Meyer

**Affiliations:** a Université de Lyon, INSA-Lyon, Université Claude Bernard Lyon 1, CNRS, UMR5240, MAP, Lyon, France; Génomique Métabolique, Genoscope, Institut Francois Jacob, CEA, CNRS, Université Évry, Université Paris-Saclay

**Keywords:** DNA supercoiling, transcriptional regulation, quantitative modeling, discriminator, stress response, evolution, biophysics, computational biology

## Abstract

DNA supercoiling acts as a global transcriptional regulator that contributes to the rapid transcriptional response of bacteria to many environmental changes. Although a large fraction of promoters from phylogenetically distant species respond to superhelical variations, the sequence or structural determinants of this behavior remain elusive. Here, we focus on the sequence of the “discriminator” element that was shown to modulate this response in several promoters. We develop a quantitative thermodynamic model of this regulatory effect, focusing on open complex formation during transcription initiation independently from promoter-specific regulatory proteins. We analyze previous and new expression data and show that the model predictions quantitatively match the *in vitro* and *in vivo* supercoiling response of selected promoters with mutated discriminator sequences. We then test the universality of this mechanism by a statistical analysis of promoter sequences from transcriptomes of phylogenetically distant bacteria under conditions of supercoiling variations (i) by gyrase inhibitors, (ii) by environmental stresses, or (iii) inherited in the longest-running evolution experiment. In all cases, we identify a robust and significant sequence signature in the discriminator region, suggesting that supercoiling-modulated promoter opening underpins a ubiquitous regulatory mechanism in the prokaryotic kingdom based on the fundamental mechanical properties of DNA and its basal interaction with RNA polymerase.

**IMPORTANCE** In this study, we highlight the role of the discriminator as a global sensor of supercoiling variations and propose the first quantitative regulatory model of this principle, based on the specific step of promoter opening during transcription initiation. It defines the predictive rule by which DNA supercoiling quantitatively modulates the expression rate of bacterial promoters, depending on the G/C content of their discriminator and independently from promoter-specific regulatory proteins. This basal mechanism affects a wide range of species, which is tested by an extensive analysis of global high-throughput expression data. Altogether, ours results confirm and provide a quantitative framework for the long-proposed notion that the discriminator sequence is a significant determinant of promoter supercoiling sensitivity, underpinning the ubiquitous regulatory action of DNA supercoiling on the core transcriptional machinery, in particular in response to quick environmental changes.

## INTRODUCTION

Bacteria encounter rapid changes of environmental conditions (availability of nutrients, physical or chemical stresses) to which they respond by quick and global modifications of their transcriptional program. Inspired by early studies, current mechanistic models of this regulatory action are mostly based on transcription factors (TFs) that bind at specific promoters and interact with RNA polymerase (RNAP). However, more than half of Escherichia coli promoters are not targeted by any known TF (1), and entire organisms are even almost devoid of them (2, 3) but nonetheless exhibit a complex regulation. Global transcriptional control has been further explained by variations in RNAP composition (sigma factors [4]) or abundance (5) depending on growth conditions as well as RNAP-binding regulatory molecules such as ppGpp (6).

Besides this variability of the transcription machinery, the physical state of the DNA template itself is subject to cellular control through DNA supercoiling (SC), i.e., the over- or underwinding of the double helix by the action of topoisomerase enzymes and architectural proteins (7–9). In bacteria, the chromosome is maintained at a negative SC level by the action of the DNA gyrase, which changes in response to environmental cues (9). This level was soon discovered to affect the expression of many promoters both *in vitro* and *in vivo* (10–14). Mechanistic studies showed that, besides modulating the binding of regulatory proteins (15), it could influence the activity of RNAP itself and, thus, could act as a global transcriptional factor (7–9). Accordingly, whole-genome analyses of the transcriptional response to DNA relaxation induced by gyrase inhibitors exhibited a broad response, providing lists of “supercoiling-sensitive genes” (3, 16–19).

In spite of its importance, no sequence or structural signature was ever clearly identified in support of the latter property. A possible reason is that SC affects transcription at many successive steps of the process, e.g., open complex formation (20, 21), promoter escape (10), elongation, and termination (22), and their combined action eluded the identification of simple determinants of supercoiling sensitivity. Additionally, transcription in turn affects the local level of SC (23), and, consequently, the response of a given promoter depends quite strongly on its genomic and physiological context (24, 25). Altogether, the complexity of the interaction between SC and transcription explains why there still are no models able to predict, even qualitatively, the response of a given promoter to variations of SC (9). The development of such predictive models is highly desirable considering the universality of superhelical variations in the prokaryotic kingdom.

One particular mechanism identified early as a putative strong factor in this response occurs at the step of open complex formation during transcription initiation (26). The unwinding of DNA strongly facilitates its denaturation and, thus, the formation of the “transcription bubble” by RNAP (11). Since this constraint affects all promoters, it may have a widespread effect on gene expression, yet the question then arises of how it may lead to transcriptional regulation, i.e., the selective activation/repression of a subset of promoters by global SC variations. An important observation was made when analyzing several stable RNA promoters as well as the *fis* promoter, which are both strongly SC sensitive and subject to stringent control (20, 27–30). Both properties are correlated with the presence of a G/C-rich discriminator sequence located between the −10 element and the transcription start site (TSS) (31), which is denatured in the open complex. The discriminator has a variable length of 5 to 8 nucleotides (nt) and does not harbor any consensus sequence but is bound by the *σ*1.2 domain of RNAP (32). Thus, it was postulated that the unusually high G/C content of these promoters affects the formation and stability of the open complex, which may then be modulated by SC, in contrast to mutant promoters containing an A/T-rich discriminator (20, 21, 30). However, it is not yet clear if this regulation mechanism is a specificity of some unusually G/C-rich promoters or a general regulatory principle by which SC quantitatively modulates the expression rate of bacterial promoters in a global and predictable manner.

In this paper, we consider the latter hypothesis and propose the first quantitative model of this mechanism, based on the free energy required to open the transcription bubble and related to the G/C content of the discriminator sequence. We show that it quantitatively recapitulates the *in vitro* and *in vivo* SC response of several promoters with mutant discriminator sequences, where the specific effect of this mechanism can be distinguished from other regulatory contributions of SC. Given its potentially broad regulatory effect, we then develop a statistical analysis of genome-wide expression data obtained after DNA relaxation by gyrase inhibitors and show that the discriminator indeed emerges as a primary location of global promoter selectivity under these conditions. We show that this sequence determinant is robustly detected in a series of phylogenetically distant bacterial species, and finally, we analyze this contribution under physiologically relevant conditions involving SC changes, induced either transiently in response to environmental stress or inheritably in the longest-running evolution experiment. Altogether, this study highlights the role of the discriminator, previously observed in a few promoters, as a global sensor of SC variations that acts independently from promoter-specific regulatory proteins and according to a predictive rule inscribed in its physical properties.

## RESULTS

### Regulatory effect of the discriminator sequence in stable RNA promoters.

**FIG 1:**
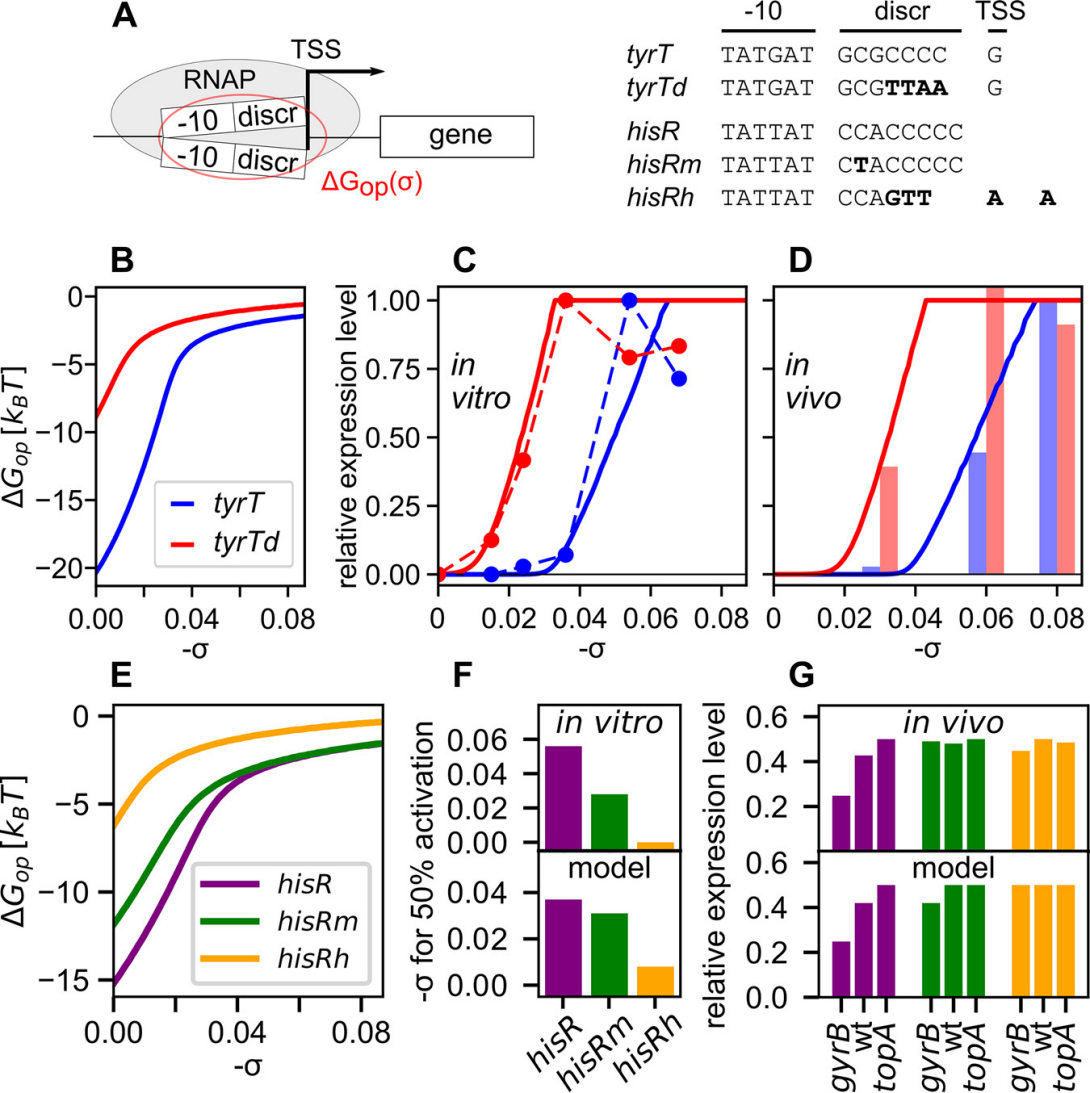
(A) Sequences from wild-type *tyrT* and *hisR* promoters, the mutant *tyrTd* promoter with A/T-rich discriminator (21), and the mutants *hisRm* and *hisRh*, with 1 and 5 substitutions, C/G→A/T, in the discriminator (20). For *hisRh*, a shift in the transcription start site (TSS) (3 nt upstream) was observed. (B) Transcription bubble opening free energies of *tyrT* and *tyrTd* promoters, computed from a thermodynamic model of DNA (see the text). (C and D) Transcription model predictions (solid lines) compared to the *in vitro* (dots) (C) and *in vivo* (bars) (D) expression data from reference 21. Data and computed values of the *tyrT* promoter are shown in blue, and those of *tyrTd* are in red. (E) Transcription bubble opening free energies of *hisR*, *hisRm*, and *hisRh* promoters. (F and G) Transcription model predictions compared to the *in vitro* (F) and *in vivo* (G) expression data from reference 20. Data and computed values of the *hisR*, *hisRm*, and *hisRh* promoters are shown in purple, green, and orange, respectively.

We first developed a quantitative model of SC-dependent transcriptional regulation based on the discriminator sequence. Negative SC destabilizes the double helix and facilitates the melting of the transcription bubble during open complex formation, which encompasses this sequence as shown in Fig. 1A. The melting energy is computed in Fig. 1B for the *tyrT* promoter (Fig. 1A) of the tyrosine tRNA operon, using a physical model of DNA denaturation (see Materials and Methods). Based on that curve, variations of the SC level should then directly affect the opening facility of promoters and, thus, their expression, and such a dependence was indeed observed for the *tyrT* promoter (blue) in both *in vitro* (Fig. 1C) or *in vivo* (Fig. 1D) transcription assays (21) (the *in vivo* SC levels are taken from reference 17). Further, the DNA denaturation energy is known to be strongly dependent on the proportion of G/C bases, and while the A/T-rich sequence of the −10 hexamer is relatively constrained due to its role in promoter recognition by the sigma factor, replacing four C/G by A/T nucleotides in the discriminator (*tyrTd* mutant) indeed strongly shifts the opening curve to the left (Fig. 1B, red curve), i.e., favors DNA opening already at weaker SC levels. Strikingly, the resulting transcriptional activation curves (Fig. 1C and D) closely follow the thermodynamic predictions.

We propose a thermodynamic model of this regulation step, based primarily on the promoter DNA opening curves (Fig. 1B), which is described in detail in Materials and Methods. It involves a single unknown parameter, representing opening assistance by RNAP, which was fitted on the data of Fig. 1 and kept constant henceforth for all promoters (thus neglecting the sequence dependence of the interaction of the discriminator with RNAP). The model reproduces most features of *in vitro* and *in vivo* activation curves of the analyzed promoters based on *tyrT* (solid lines in Fig. 1C and D). We tested it further using a similar data set collected independently based on the promoter of *hisR*, the histidine tRNA of S. enterica (20). *In vitro* (Fig. 1F), the expression increases with negative SC, both in the WT and in mutant promoters of variable G/C richness in the discriminator, closely following the DNA opening curves of the associated sequences (Fig. 1E), and, thus, are approximately reproduced by the model without any parameter adjustment. *In vivo*, only the native promoter was affected (Fig. 1G) in topoisomerase mutant strains exhibiting a global SC shift either in the direction of DNA relaxation (*gyrB* mutant) or SC increase (*topA*). This feature was reproduced using the experimentally measured SC levels of these strains (33), suggesting that the two A/T-rich mutant promoters have reached a plateau where the denaturation energy and, hence, the expression level is almost independent of SC.

The model was kept voluntarily as simple as possible, since this mechanism is only one of the multiple steps by which SC affects transcription (as further developed in Discussion) and a reduced number of adjustable parameters was a key advantage. The approximations used in the modeling of this specific step as well as those other contributing factors may explain the slight discrepancies with the data (see details in Materials and Methods), but the clear overall agreement supports the notion that the proposed mechanism is the primary contributor in the SC sensitivity of promoters controlled by the discriminator sequence.

### Validation of model predictions on mutant mRNA promoters.

**FIG 2:**
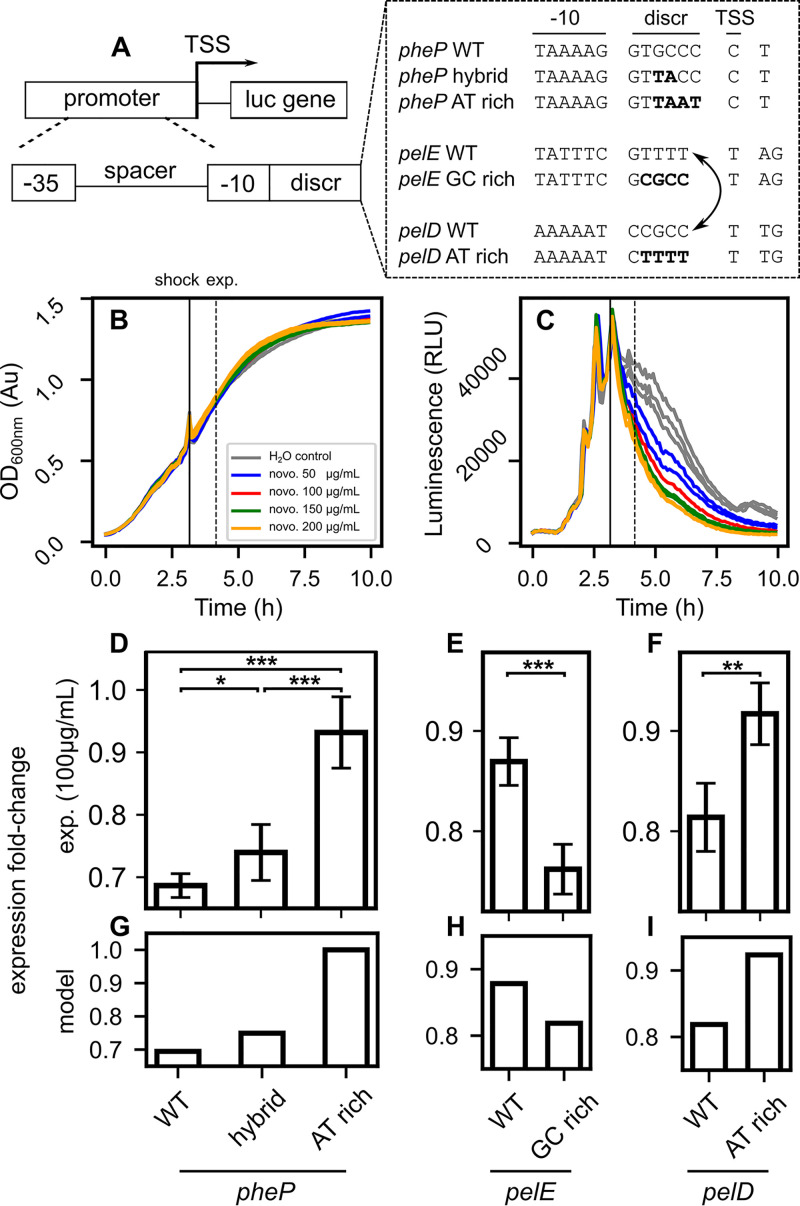
DNA relaxation response of promoters with mutated discriminators. (A) Promoter sequences were derived from *pheP* (E. coli) and *pelD*-*pelE* (*D. dadantii*), with mutated discriminators of various G/C contents. (B) Bacterial growth monitored in a microplate reader (E. coli bacteria carrying plasmids with *pheP* hybrid promoter in rich medium). A novobiocin shock was applied in mid-exponential phase (different sublethal concentrations are shown). The slight increase at shock time is an optical artifact due to the opening of the recorder. (C) Expression of the *pheP* hybrid promoter monitored by luminescence (see all raw data points in Fig. S2). (D) Expression fold changes in response to relaxation computed 60 min after novobiocin shock (100 μg/ml) in *pheP*-derived promoters. As expected, the repression factor reduces with increasing A/T%. (E and F) The DNA relaxation response of *pelE* (E) and *pelD* (F) are reversed when a tetranucleotide is swapped between their discriminators, with low and high G/C content, respectively. (G) Expression fold changes in response to relaxation predicted by the model reproduce the experimental observations on *pheP*-derived promoters as well as *pelE* (H)- and *pelD* (I)-derived promoters, assuming a weak relaxation compatible with the observed repression levels (see the text). Error bars represent 95% confidence intervals, and stars indicate the level of statistical significance (see Materials and Methods).

We then further tested the validity of the model by measuring the regulatory effect (expression fold change) of superhelical variations on mutant promoters of protein-coding genes with different features. Two families of synthetic promoters were constructed (Fig. 2A; see also Table S1 in the supplemental material). The first family is based on the *pheP* promoter of E. coli, which is SC sensitive (16, 17) and not regulated by any identified TF (1) and is an interesting candidate for our regulation mechanism based on the basal interaction with RNAP; these promoters were analyzed in LB medium, where gyrase activity is high (7). The second family is made of the paralogous virulence genes *pelD-pelE* of the enterobacterial phytopathogen Dickeya dadantii, encoding similar pectinolytic enzymes; in contrast to *pheP*, these genes exhibit a high regulation complexity, with more than 10 identified TFs, and both are supercoiling sensitive (34) but harbor different discriminators. These promoters were analyzed in minimal medium, which is closer to their physiologically relevant conditions (plant apoplast).

10.1128/mSystems.00978-21.8 TABLE S1 List of synthetic promoter sequences with mutated discriminators and plasmids and primers used to construct *D. dadantii* 3937-derivative strains with *pelD-luc* and *pelE-luc* transcriptional fusions in the *pelA-pelE* intergenic region of the chromosome. Download Table S1, PDF file, 0.04 MB. Copyright © 2021 Forquet et al. 2021 Forquet et al. https://creativecommons.org/licenses/by/4.0/ This content is distributed under the terms of the Creative Commons Attribution 4.0 International license.

10.1128/mSystems.00978-21.3 FIG S2 Bacterial growth and mutant promoter expression monitored in E. coli in a microplate reader (raw data). For all promoters, the left panel shows bacterial growth measured by OD_600_ and the right panel shows the expression level measured by luminescence. (A) E. coli bacteria carrying plasmids with *pheP* WT promoter in rich medium. (B) *pheP* AT-rich promoter. (C) *pelE* WT promoter in minimal medium with only 100 μg/ml novobiocin concentration tested. (D) *pelE* GC-rich promoter. (E) *pelD* WT promoter. (F) *pelD* AT-rich promoter. The OD_600_ discrepancies at shock time or after several hours in some cases (D, E, and F) are optical artifacts due to the opening of the recorder and/or the formation of sediments disrupting the measurements (the luminescence does not vary correspondingly among replicates). Download FIG S2, PDF file, 0.2 MB. Copyright © 2021 Forquet et al. 2021 Forquet et al. https://creativecommons.org/licenses/by/4.0/ This content is distributed under the terms of the Creative Commons Attribution 4.0 International license.

Promoters were fused on plasmids in front of a luciferase reporter gene (Fig. 2A), and their expression was analyzed in E. coli cells in a microplate reader after treatment by novobiocin, which relaxes the chromosomal DNA by inhibiting gyrase and, to a lesser extent, topoisomerase IV (35). The employed plasmids are well established as reflecting the average SC level of the chromosome (36), in particular during DNA relaxation by novobiocin (34, 37, 38).

We first checked that the presence of the plasmids did not affect bacterial growth and that the expression patterns of two promoters as well as their response to novobiocin were consistent when inserted either in plasmid-borne or in chromosomal luciferase fusions (Fig. S1). These observations match previous similar comparisons involving other promoters and plasmids (30, 39) and confirm that the reduction in luminescence observed following the shock (raw data in Fig. S2) is due to SC-dependent transcriptional regulation rather than plasmid-specific effects. We then compared the relative effect of the novobiocin shock on the different plasmid-borne promoters. For the *pheP*-derived promoters (Fig. 2D), we found that the expression fold change (treated versus nontreated wells) was strongest for the native G/C-rich promoter and significantly reduced for the hybrid promoters (with two mutated nucleotides in the discriminator), whereas the A/T-rich discriminator (with four mutated nucleotides) was weakly sensitive to DNA relaxation. Thus, as already suggested *in vitro* with the *hisR* promoter (Fig. 1F), the SC sensitivity is progressively modulated by the discriminator G/C% *in vivo*. Similarly, swapping four nucleotides between the discriminators of *pelE* and *pelD* (Fig. 2E and F) strikingly reversed their response to DNA relaxation. The relatively modest (but highly significant) repression levels are partly due to a buffering effect of the reporter system. Since the exact SC levels are not known under these conditions, we fitted the data using three adjustable parameters (an initial SC level for each growth medium and a common relaxation magnitude), which allowed us to reproduce the results with good accuracy (using, as expected, a stronger SC level in rich medium; Fig. 2G to I and Materials and Methods). Note that the direction of the promoters’ predicted response is inscribed in their sequences and therefore is qualitatively robust when the exact value of these parameters is varied.

10.1128/mSystems.00978-21.2 FIG S1 Bacterial growth and promoter expression monitored in a microplate reader for *pelE-luc* and *pelD-luc* transcriptional fusions either inserted into *D. dadantii* chromosome or incorporated into pUCTer-*luc* plasmids and transformed into *D. dadantii* cells. (A) Bacterial growth measured by OD_600_ and *pelE* expression level measured by luminescence in minimal medium. A novobiocin shock was applied in mid-exponential phase (100 μg/ml concentration). (B) Same for *pelD*. The patterns of bacterial growth, promoter expression, and response to novobiocin are consistent between the two systems (chromosomal fusions versus plasmid-borne fusions) for both *pelE* and *pelD*. (C) Absolute expression levels for *pelE* promoter in absence of shock. As expected, the multicopy plasmid-borne fusion is expressed at a higher level than the chromosome-borne fusion. (D) Comparison of expression fold changes in response to relaxation for *pelE* promoter, demonstrating a quantitatively identical effect of novobiocin in the two constructions, in spite of the difference of absolute expression. This observation confirms that the fold-changes observed on the plasmid-borne constructions are indeed due to a regulatory effect of DNA relaxation (identical in the chromosome and in the plasmid) rather than a plasmid-specific effect (copy number variation, etc.). The relaxation activity of novobiocin was previously demonstrated in *D. dadantii* cells on the plasmids employed (34). (E and F) Same for *pelD* promoter. (C to F) Computed from the final luminescence time points in panels A and B. Download FIG S1, PDF file, 0.1 MB. Copyright © 2021 Forquet et al. 2021 Forquet et al. https://creativecommons.org/licenses/by/4.0/ This content is distributed under the terms of the Creative Commons Attribution 4.0 International license.

These results show that the effect of the discriminator on the SC sensitivity is not specific to G/C-rich ones (such as those of stable RNAs or *fis*) but is a quantitative effect that is progressively modulated by the G/C% and equally affects promoters with a naturally low G/C%, such as *pelE*, as expected from our modeling. It affects promoters of diverse biological functions and regulation complexities and is detectable under different physiological conditions (rich versus minimal medium). Based on these observations on a few selected promoters, and since the proposed mechanism of open complex formation is involved in RNAP-promoter interaction independently from additional regulatory proteins, we now enlarge the scale of the analysis to entire genomes.

### The discriminator is a primary location of promoter selectivity by DNA relaxation.

We first looked at the variability of discriminator G/C contents among mRNA promoters in various species based on available TSS maps (Fig. S3). These distributions are wide, and like *pheP* and *pelD*, a large class of promoters have G/C-rich discriminators. Based on the previous analysis, we hypothesized that such promoters would be more repressed by a DNA relaxation induced by gyrase inhibitors than those harboring an A/T-rich discriminator. However, in contrast to the mutation data described above, here the compared promoters differ by many additional factors beyond their discriminator sequence (upstream and downstream sequences, genomic context, binding of regulatory proteins, etc.), which may contribute to their supercoiling response; therefore, we looked for a statistical relation rather than a prediction valid for all analyzed promoters.

10.1128/mSystems.00978-21.4 FIG S3 Variability of promoter discriminator A/T content in the investigated organisms. Proportion of promoters depending on their A/T content in the discriminator defined as the sequence of variable length between −10 element and +1 positions (A) or the 5-nt sequence centered around position −2 (B) used throughout the manuscript. For E. coli, *S.* Typhimurium, *D. dadantii*, and M. pneumoniae, only σ^70^ promoters were considered, whereas only *σA* promoters were considered for *S. elongatus*. All promoters were aligned at their −10 site, except for *S. elongatus* and M. pneumoniae for which promoters were aligned at their annotated TSS, and the signal shifted 4 nt in the case of M. pneumoniae. The distributions are similar with the two methods. Download FIG S3, PDF file, 0.09 MB. Copyright © 2021 Forquet et al. 2021 Forquet et al. https://creativecommons.org/licenses/by/4.0/ This content is distributed under the terms of the Creative Commons Attribution 4.0 International license.

**FIG 3:**
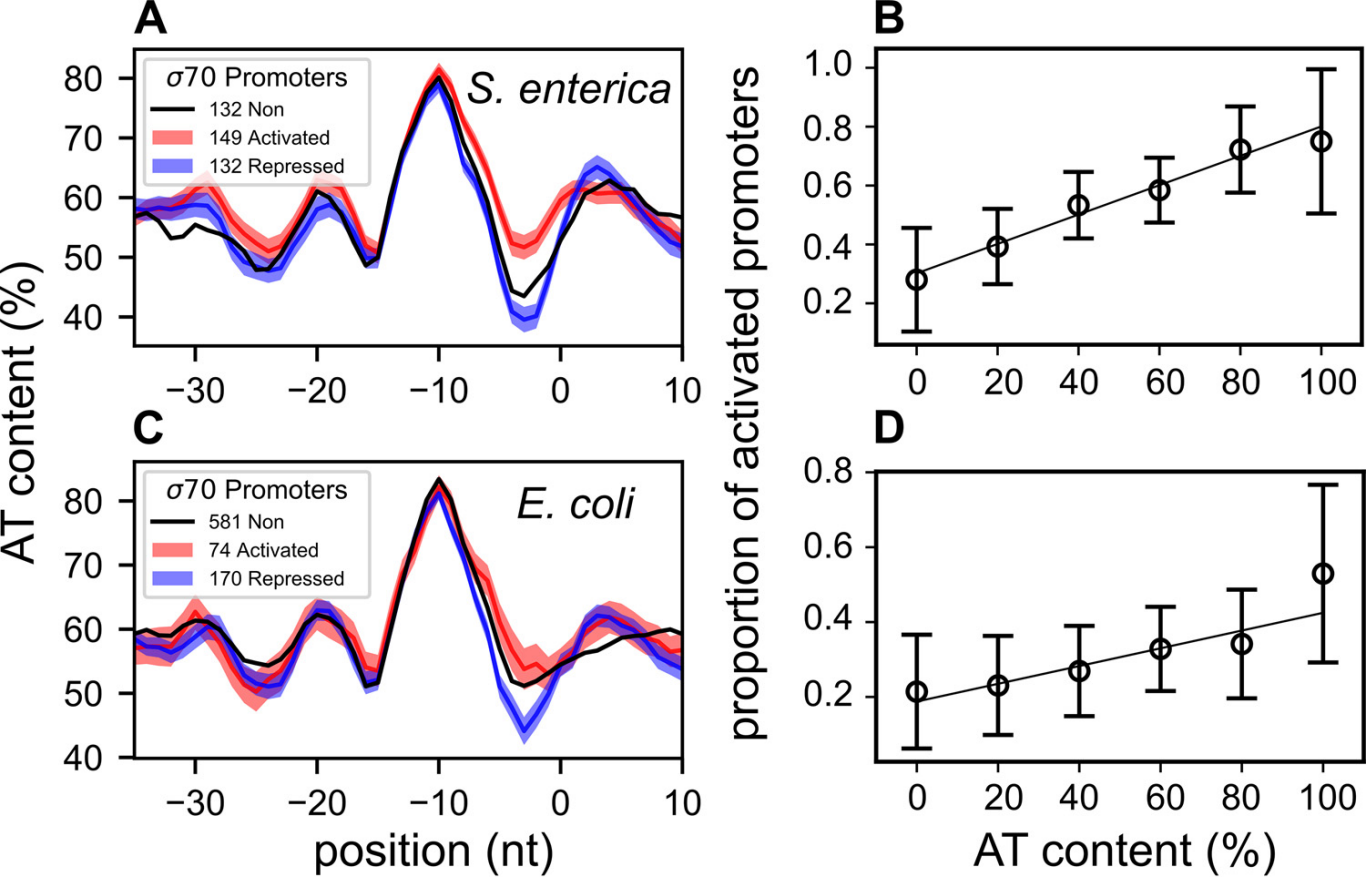
Genome-wide relation between discriminator sequence and promoter selectivity during DNA relaxation. (A) Average A/T% profiles of S. enterica σ^70^ promoters along 5-nt-centered windows, depending on their response to novobiocin-induced chromosomal relaxation (19) (activated, significantly upregulated promoters; repressed, significantly downregulated promoters; non, not significantly affected). Colored-shaded areas represent ± one standard error (67% confidence intervals). The profiles are very similar except in the discriminator region (between −10 and +1 positions). (B) Proportion of activated promoters among those responsive to DNA relaxation, depending on their A/T% in a 5-nt window centered around position −2. Error bars represent 95% confidence intervals. The resulting linear regression is highly significant (*P* < 10^−4^). (C) Average A/T% profiles of E. coli σ^70^ promoters depending on their response to norfloxacin-induced DNA relaxation (LZ54 versus LZ41 strains [17]). The resulting pattern is very similar to that observed in S. enterica in spite of strong differences in protocols. (D) Same as panel B but for the E. coli data (*P* = 0.011).

We aligned all σ^70^ promoters of Salmonella enterica and looked at their average A/T% profile (Fig. 3A) depending on their response 20 min after a novobiocin shock (19). Strikingly, although this content exhibits a characteristic nonuniform pattern along the promoter (with an expected peak at the −10 element), the signals of the two groups of promoters are indistinguishable everywhere except in the region between −10 and +1, precisely where we expected the observed difference ($P<10^{-5}$ around position −2; Table S2). This observation, obtained independently from the mutation studies described above, confirms that the discriminator region is a primary location of selectivity for the relaxation response. As a comparison, no significant difference is detected at the −10 element, suggesting that this selectivity is not related to a difference in sigma factor usage. Further, classifying the promoters based on their discriminator sequence composition (Fig. 3B) exhibits a clear and highly significant (approximately linear) effect on the proportion of activated promoters (correlation *P* $<10^{-4}$).

10.1128/mSystems.00978-21.9 TABLE S2 Compilation of investigated species, conditions, and results. The SC change refers to the direction of SC variation induced by the condition (−, DNA relaxation; +, increase in negative SC). The correlation condition from *S. elongatus* corresponds to the phasing of gene expression in the SC circadian oscillation and provides an indirect proxy of gene response to SC relaxation (42). For the stress conditions, the protocols used in the SC assays usually differ from those used in the transcriptomic experiments and, thus, only give a semiquantitative estimate of the levels involved in the observed regulatory response. Under all conditions, genes were considered significantly activated/repressed based on a threshold of 0.05 on the adjusted *P* value, except for the evolution data (0.3). For heat/cold shock, and *S. elongatus*, *P* values were not provided and were replaced by thresholds on log_2_-fold-change values (0.5) to generate subsets of act/rep genes of comparable sizes as in other datasets. A/T% contents are compared with Student tests, in 5-nt windows centered at position −2 in the discriminator region. The sensitivity gain is computed as the increase in the proportion of accurately predicted genes (among significantly activated or repressed genes) compared to a null (random) model. Download Table S2, PDF file, 0.08 MB. Copyright © 2021 Forquet et al. 2021 Forquet et al. https://creativecommons.org/licenses/by/4.0/ This content is distributed under the terms of the Creative Commons Attribution 4.0 International license.

### A robust relation observed across phylogenetically distant bacterial phyla.

Since the investigated mechanism relies on highly conserved molecular actors, RNAP and topoisomerases, it might affect a broad range of bacterial species. We therefore tested the validity of our observations in all organisms where a transcriptome obtained after DNA relaxation was available together with an accurate TSS map (from independent studies). The list of references of the employed data is summarized in Table S2, and the table of detailed promoter sequences is in Table S3.

10.1128/mSystems.00978-21.10 TABLE S3 List of detailed promoter sequences for (A) E. coli, (B) *S.* Typhimurium, (C) *D. dadantii*, (D) *S. elongatus*, and (E) M. pneumoniae. Genome-wide TSS maps were collected from the literature (Table S2), and a scan for promoter motifs was conducted with bTSSfinder (60), imposing each TSS position at the experimentally determined nucleotide. For M. pneumoniae, we used position weight matrices from E. coli sigma factors and indicated all promoters identified by the bioinformatic analysis (although the sigma factors in this species differ from those of E. coli; this information is not used in the analysis where all promoters are aligned at the TSS). Download Table S3, XLSX file, 0.3 MB. Copyright © 2021 Forquet et al. 2021 Forquet et al. https://creativecommons.org/licenses/by/4.0/ This content is distributed under the terms of the Creative Commons Attribution 4.0 International license.

Transcriptomic data were obtained in E. coli with DNA microarrays after norfloxacin shock in two alternate topoisomerase mutant strains (40), resulting in a strong magnitude of DNA relaxation (17). In spite of strong differences in the experimental protocol compared to the S. enterica data set, the obtained pattern is remarkably similar (Fig. 3C and D). Importantly, whereas in the first experiment (treated versus nontreated cells) this pattern might include contributions from SC-independent drug response pathways, here the two compared samples received exactly the same treatment, and any such unwanted contribution should not be apparent. The slightly weaker observed effect might also be due to the lower sensitivity of the employed transcriptomic technology.

**FIG 4:**
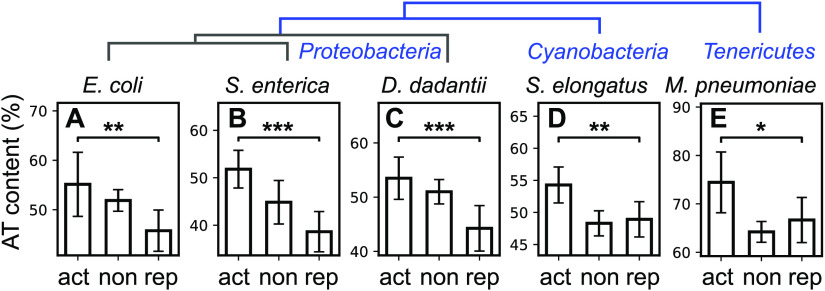
Robust statistical relation between discriminator A/T% and promoter’s response to DNA relaxation (act, activated; non, no significant variation; rep, repressed) is observed in phylogenetically distant bacterial species: (A) E. coli (*P = *0.010, relaxation by norfloxacin in LZ54 versus LZ41 mutant strains) (17); (B) *S.* Typhimurium (*P* < 10^−5^); (C) *D. dadantii* (*P* < 10^−3^); (D) *S. elongatus* (*P = *0.004); and (E) M. pneumoniae (*P = *0.029). In enterobacteria, only σ^70^ promoters were considered and were aligned at the −10 element. In the two other species, all promoters were aligned at their annotated TSS, resulting in a poorer definition of the signal and positional shifts. A/T% are computed in a 5-nt window centered around position −2 in the discriminator region, except for *S. elongatus* (position +4 after the TSS). Error bars represent 95% confidence intervals, and stars indicate the level of statistical significance (see Materials and Methods). A schematic phylogeny is depicted above.

In *D. dadantii*, the response to relaxation by novobiocin was monitored in minimal medium (25) based on identified gene promoters (41). It exhibits the same pattern (Fig. 4C, more details are given in Fig. S4) as in E. coli (Fig. 4A) and S. enterica (Fig. 4B), suggesting that the investigated mechanism is valid for a broad range of enterobacteria of diverse lifestyles. Note that in Fig. 4 and later figures, genes not significantly affected by DNA relaxation were shown for qualitative comparison purpose but are heterogeneous among data sets and should not be used for rigorous statistical comparisons (heterogeneous and unknown false-negative rates).

10.1128/mSystems.00978-21.5 FIG S4 Relation between discriminator sequence and response to SC variations in all investigated conditions and organisms. For each expression dataset, the left panel corresponds to average A/T% profiles of promoters along 5-nt-centered windows depending on their transcriptomic response, while the right panel corresponds to the proportion of activated promoters depending on their A/T% in a 5-nt window around position −2 in the discriminator region (except for *S. elongatus*, position +4). The *P* value of the resulting linear regression is indicated. Act refers to significantly upregulated promoters, Rep to significantly downregulated promoters, Non to not significantly affected promoters. Colored shaded areas represent 67% confidence intervals, while error bars represent 95% confidence intervals. *Δσ*> 0 refers to DNA relaxation, whereas *Δσ*< 0 refers to an increase in negative SC. Data are for (A) E. coli, (B) *D. dadantii*, (C) *S. elongatus*, and (D) M. pneumoniae. In enterobacteria (A and B), only σ^70^ promoters were considered, and aligned at the −10 element, whereas in the two other species (C and D), all promoters were aligned at their annotated TSS, resulting in a poorer definition of the signal and positional shifts. All references are provided in Table S2, and detailed promoter sequences are in Table S3. Download FIG S4, PDF file, 0.4 MB. Copyright © 2021 Forquet et al. 2021 Forquet et al. https://creativecommons.org/licenses/by/4.0/ This content is distributed under the terms of the Creative Commons Attribution 4.0 International license.

Data were also available for two species of drastically larger evolutionary distance, the cyanobacterium Synechococcus elongatus and the small tenericute Mycoplasma pneumoniae. In these species, because the sigma factors differ from those of enterobacteria, the alignment of promoter elements was obtained with a poorer definition (promoters aligned at the TSS; see Materials and Methods). We nevertheless looked for sequence signatures comparable to those observed previously. In Synechococcus elongatus, where SC was shown to be a major determinant of circadian oscillatory genomic expression (42), the transcriptomic response to DNA relaxation was not monitored directly, but the phasing of gene expression in this oscillation can be used as an indirect proxy of this response (42), although many other metabolic signals may be equally correlated and could contribute to this signal. As a result of the analysis, a similar difference of discriminator sequence was detected as in enterobacteria (Fig. 4D) of slightly lower magnitude and at a position slightly shifted after the TSS (Fig. S4), possibly due to the poorer resolution of the analysis and the additional regulatory mechanisms involved. In the small tenericute Mycoplasma pneumoniae, in which transcriptional regulation is poorly understood due to the quasi-absence of TFs (43), the response to novobiocin was also monitored (3). Although the signal is also weakened by the spatial resolution and by the lower number of promoters, it is still significant at the same location in the discriminator as in enterobacteria (Fig. 4E).

Altogether, the same signature is robustly and consistently observed in available data sets obtained after DNA relaxation in enterobacteria, and, with limitations due to the available definition of promoters and heterogeneity of the analyzed data, in two phylogenetically distant species that differ widely from the others in terms of lifestyle and average G/C content (in particular, M. pneumoniae has very few promoters with strongly G/C-rich discriminators; Fig. S3). These results suggest that the ancestral infrastructural constraint of DNA opening, coupled with the conserved activity of topoisomerases, indeed underpins a global regulatory mechanism throughout the prokaryotic kingdom.

### Global response to stress conditions and inheritable supercoiling variations.

While sublethal antibiotic shocks are the classical method of choice to specifically induce rapid DNA relaxation (9, 44), under natural conditions the latter is rather triggered by sudden changes of environmental conditions, especially by physicochemical stress factors like temperature, acidity, oxidative agents, etc. The resulting rapid SC variations were found to be conserved even in phylogenetically distant species, e.g., increase of negative SC by cold shock, DNA relaxation by heat shock, or oxidative stress (9). We therefore tested if the sequence signature expected from the analysis described above could be detected in published transcriptomic data, although other stress-specific pathways contribute to the response and might hide this signature. Such data were obtained under various conditions (9); in the following, we focus our analysis on temperature and oxidative stress, where (i) the associated SC variations are well documented; (ii) there is no indication of ppGpp induction (see Discussion); and (iii) under each condition, two independent data sets were available and gave similar results.

**FIG 5:**
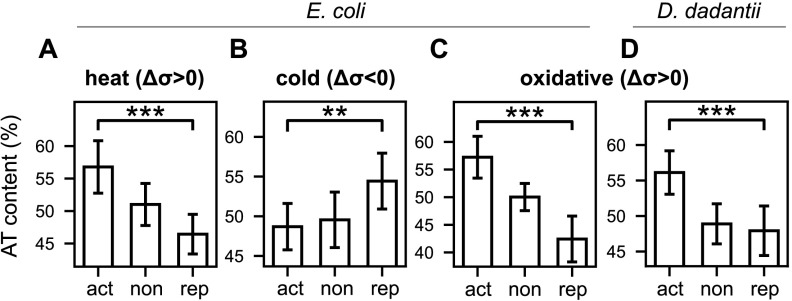
Relation between discriminator sequence and response to SC variations induced by environmental stress conditions (act, activated; non, no significant variation; rep, repressed). A/T% are computed in a 5-nt window centered around position −2 in the discriminator region. (A) During heat shock in E. coli (45), triggering a transient DNA relaxation (*Δσ*> 0), activated promoters have discriminators with higher A/T% than repressed ones (*P* < 10^−5^), as expected from the presented model. (B) In a cold shock in E. coli (46) inducing an opposite SC variation (increase in negative SC, *Δσ*< 0), the relation is reversed, with a preference of G/C-rich discriminators among activated promoters (*P = *0.007), as we expected. (C and D) Same as panels A and B but during an oxidative shock in E. coli (47) inducing DNA relaxation (*σ* > 0, *P* < 10^−7^) (C), and in *D. dadantii* (*P* < 10^−4^) (D) (18), where the shock was shown to induce the same SC response (34), showing the conservation of the mechanism. Error bars represent 95% confidence intervals, and stars indicate the level of statistical significance (see Materials and Methods).

Heat and cold shocks both put the bacteria under stress while affecting the SC level in opposite directions (relaxation and overtwisting, respectively; Table S2). The analysis of the corresponding transcriptomic data sets (45, 46) clearly confirms the expectations, with G/C-rich discriminators being repressed and activated with a linear dependence in the sequence content (Fig. 5A to C; see also the spatial patterns in Fig. S4). Similar signals were observed based on independent data sets obtained under the same conditions (47 and data not shown). In the case of oxidative stress (induced by H_2_O_2_) associated with DNA relaxation, the response was analyzed in the enterobacteria E. coli and *D. dadantii* (18, 47), where the pattern is indeed very similar and matches the expectations. Altogether, this analysis suggests that, beyond stress-specific regulation pathways mediated by dedicated regulatory proteins, the SC variations induced under these conditions play a direct role in the resulting global reprogramming of gene expression by modulating the RNAP-promoter interaction through the discriminator sequence. Under other stress conditions (osmotic or acidic stress) that we analyzed, the signal was species or data set dependent (data not shown), suggesting that other regulation mechanisms play a stronger role.

**FIG 6:**
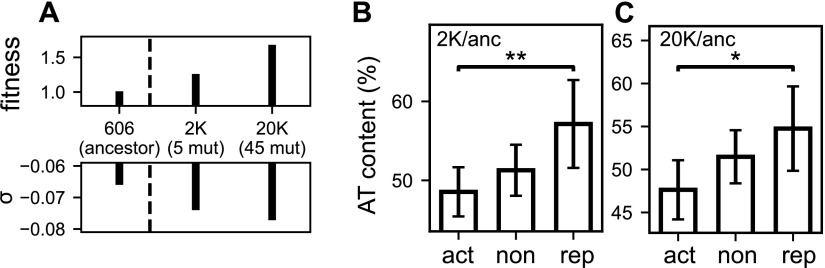
(A) In the longest-running evolution experiment (48), two point mutations naturally acquired by E. coli (49) induced successive increases of negative SC, one in *topA* before 2,000 generations (among the five observed) and one in *fis* before 20,000 generations (among the 45 observed), and are associated with fitness gains through modifications of global gene expression. Adapted from reference 25. (B) Proportion of A/T content in the discriminator (5-nt window centered around position −2) of promoters activated, repressed, or not significantly affected in the evolved strain 2K compared to the ancestor. As expected from our modeling for an increase in negative SC, activated promoters with G/C-rich discriminators are more activated (*P = *0.005). (C) In the 20K evolved strain, the same difference is observed (*P = *0.011), although less significant, possibly due to many other mutations affecting the regulatory network. Error bars represent 95% confidence intervals, and stars indicate the level of statistical significance (see Materials and Methods).

Finally, we address the question of whether the investigated mechanism is involved not only in transient responses but also in inheritable modifications of the expression program. In the longest-running evolution experiment with E. coli (48), point mutations inducing variations of the SC level were indeed quickly and naturally selected (49), as they provided substantial fitness gains that were attributed to the resulting global change of the transcriptional landscape (25). In the investigated conditions of growth in nutrient-poor medium, a first mutation (in *topA*, among 6 in total) before 2,000 generations and a second mutation (in *fis*, among 45 in total) before 20,000 generations both lead to an inheritable increase of negative SC (Fig. 6A). Based on the modeling, these mutations should predominantly enhance the expression of promoters with G/C-rich discriminators in the evolved strains. Such a tendency is indeed observed in both available transcriptomes that we analyzed, obtained either after 2,000 generations, where the signal is strongest (Fig. 6B) (*P* = 0.005), or after 20,000 generations (*P* = 0.011) (Fig. 6C, and Table S2), where 43 accumulated mutations besides these two affecting SC probably contribute to rewiring the regulatory network and blurring the signal. The detected signature suggests that the proposed biophysical regulatory mechanism not only is involved in rapid changes of gene expression but also may be used as a driving force in the evolution of genomes.

## DISCUSSION

In this work, we propose a simple thermodynamic model of open complex formation that quantitatively accounts for transcriptional regulation by SC based on the discriminator sequence. Our analysis confirmed and gave a quantitative content to the long-proposed notion that the discriminator sequence is a significant determinant of promoter supercoiling sensitivity. The statistical analysis of promoter sequences, carried out in various species and experimental conditions, highlights the widespread relevance of this mechanism in the genome-wide response to transient or inheritable variations of SC levels.

Interestingly, a global analysis of σ^70^-dependent promoter sequences in E. coli yields a significant negative statistical relation between the A/T% at the discriminator and the RNAP binding score at the −10 element (as computed from its sequence motif, Pearson’s $R=-0.18,\;P<10^{-16}$), suggesting that intrinsically attractive promoters have higher G/C-rich discriminators and, thus, are more difficult to open. This observation suggests that open complex formation is used as a general regulation mechanism for highly expressed operons, as occurs in rRNA promoters (7) (although a high affinity at the −10 element does not imply a high expression level). However, we did not observe any A/T% difference at the −10 element between promoters activated and repressed by SC (Fig. 3A and C), suggesting that high RNAP affinity and SC-mediated regulation are independent. While this study is focused on the specific role of SC, the general relation between RNAP affinity and the discriminator sequence might also involve other regulation mechanisms (including ppGpp; see below).

### Quantification and limitations of the regulatory mechanism.

A major difficulty when analyzing SC-induced regulation is that it affects the transcription process at multiple steps from the binding of regulators to the activity of RNAP itself during transcription initiation (10), elongation, and termination (22). While we focused our analysis on the discriminator sequence, the reader should keep in mind that many other mechanisms contribute to enhancing the complexity of this regulation: (i) the influence of DNA conformation on its interaction with regulatory proteins (9); (ii) competing structural transitions (denaturation, cruciform exclusion, G-quadruplex, and Z-DNA) occurring in nearby regions depending on the SC level and strongly affecting the SC response at the initiation site (50); (iii) the modulation of the effective SC level available for denaturation because of twist/writhe dynamics and local mechanical constraints imposed by regulatory proteins (9); and (iv) the heterogeneity of SC levels in different topological domains along the chromosome (51), in contrast to the approximation of a homogeneous level considered in this study. In particular, this heterogeneity was shown to depend on the local orientational organization of the genome because of the dynamic production of supercoils by elongating RNAPs. A recently proposed model of this mechanism, complementary to this study, explains a significant contribution to the transcriptional response to DNA relaxation even when all promoters are assumed to respond identically to SC variations (25). Therefore, integrating these two complementary factors of complexity, orientation-dependent heterogeneity of SC levels and sequence-dependent heterogeneity of promoter response, into a unified model is a natural objective for future studies.

These various complexity factors and others explain why, in the analyzed transcriptomic data, the effect of the discriminator sequence emerges as a statistical feature at the genomic scale rather than a predictive signal dictating the response of each individual promoter as observed in mutation studies. In particular, since a negative SC level favors the denaturation of G/C-rich as well as A/T-rich sequences (Fig. 1), this mechanism alone is insufficient to explain the existence of a class of relaxation-activated promoters, such as *gyrA-gyrB* (13). This behavior might be explained by more complex mechanisms involving the kinetics of promoter opening and escape by RNAP, where the stability of the open complex becomes unfavorable if it leads to abortive rather than processive transcription (10, 26), by thermodynamic competition with other structural transitions occurring at nearby sites (50), or by the effect of SC on the binding of transcription factors that are sensitive to the DNA tridimensional conformation (indirect readout) (15).

In spite of these limitations of our modeling, and based on the sequence signal observed in transcriptomic data, can we quantify the contribution of this specific mechanism in the genome-wide supercoiling response? To estimate this magnitude, we developed a genome-wide prediction of the relaxation-response based solely on the thermodynamic opening model developed above (independently from all other transcriptional effects of SC) and computed the proportion of accurate predictions among the observed differentially expressed genes (activation or repression). Compared to a null (random) model, this proportion is improved by around 10 to 15% of the responsive genes in the investigated relaxation and environmental stress assays (usually several hundred, representing a high statistical significance of predictive power; see details in Table S2 in the supplemental material). Considering the many alternate regulatory mechanisms by SC, for which no comparable estimates are available at the genomic scale (most of them lacking quantitative models), this proportion computed from a single step without parameter adjustment is quite notable. Additionally, it is likely underestimated because of many inaccurately annotated promoters (a single-nucleotide resolution is required but often not achieved) and may be reevaluated in the future based on more precise annotations. Note that because the total mRNA levels are normalized in transcriptomic data (predefined sequencing depth, erasing any global activation/repression effect), we introduced a comparable normalization step in the computation. As a result, a fraction of A/T-rich promoters appear to be activated by the DNA relaxation even if they are more difficult to open by RNAP (by competition with G/C-rich ones; see Fig. S6 and Materials and Methods).

10.1128/mSystems.00978-21.7 FIG S6 Model prediction of the response of S. enterica promoters to DNA relaxation. After applying the model of the first equation to all promoters, a normalization step mimics the protocol of the transcriptomic experiment. The proportion of promoters predicted as activated is then computed depending on their A/T% in a 5-nt window around position −2 in the discriminator region (same as Fig. 3B and D and Fig. S4). Download FIG S6, PDF file, 0.02 MB. Copyright © 2021 Forquet et al. 2021 Forquet et al. https://creativecommons.org/licenses/by/4.0/ This content is distributed under the terms of the Creative Commons Attribution 4.0 International license.

### Simultaneous regulation by SC and ppGpp at the discriminator.

Among various further regulatory mechanisms related to this study, the alarmone ppGpp, classically associated with the stringent (starvation) response (6), deserves special attention. In contrast to many TFs, ppGpp affects the expression of a large subset of the genome by binding RNAP in combination with the transcription factor DksA (52) and modulating the stability of the open complex (29). Its repressive effect is not dependent on a strict sequence motif but rather on the presence of a C nucleotide at position −1 (52). This regulatory mechanism presents many similarities to the one investigated here, and both are involved in the regulation of bacterial growth, raising the possibility of interplay between these two pathways (29, 53).

We first checked that the sequence signatures identified in this study were not due to a regulatory effect involving ppGpp rather than SC. It was observed that gyrase inhibition does not trigger any growth arrest (Fig. 2B) or signature of stringent response (17); accordingly, an analysis of the expression levels of genes involved in ppGpp synthesis (*gppA*, *spoT*, and *relA*) does not exhibit any significant response (3, 16–19). Thus, DNA relaxation does not trigger ppGpp production, and even if the two pathways are associated with a similar sequence signal in the discriminator, the observations made in this study are indeed due to a ppGpp-independent effect of SC.

We then carried out a sequence analysis of the promoters directly regulated by ppGpp through its binding to RNAP, as identified at the genomic scale in a recent study in E. coli (52). As expected, a strong difference in G/C% between the many promoters activated and repressed by ppGpp induction (representing 70% of σ^70^ promoters in total) is detected in the discriminator (Fig. S5A), similar to the pattern observed with DNA relaxation (Fig. 3), confirming that the two pathways affect transcription at the whole-genome scale based on similar promoter sequence determinants.

10.1128/mSystems.00978-21.6 FIG S5 Simultaneous regulation by SC and ppGpp at the discriminator. (A) Average A/T% profiles of E. coli *σ*70 promoters along 5-bp centered windows, depending on their response to ppGpp induction (activated, significantly upregulated promoters; repressed, significantly downregulated promoters; non, not significantly affected). Data are from reference 52, strain 1-2-pALS13, after 5 minutes (310 activated and 313 repressed promoters). The A/T% profiles of the promoter groups are very similar (overlapping 67% confidence intervals, visible as colored shaded areas) except in the discriminator region (between −10 and +1 positions). (B) Same in the 1-2-pALS13 strain after 10 minutes harboring a mutant RNA polymerase unable to bind ppGpp. ppGpp still induces a differential expression of around half as many genes (153 activated and 160 repressed promoters), and the difference in A/T% is still strongly present, although weaker than that in A. We suggest ppGpp-induced SC relaxation as a plausible mechanism for this regulation. Download FIG S5, PDF file, 0.06 MB. Copyright © 2021 Forquet et al. 2021 Forquet et al. https://creativecommons.org/licenses/by/4.0/ This content is distributed under the terms of the Creative Commons Attribution 4.0 International license.

While DNA relaxation does not induce ppGpp production, it was conversely shown that the induction of high levels of ppGpp by the stringent response does trigger a sharp fall in SC levels in E. coli (29). Thus, it is plausible that the strong sequence signature observed after ppGpp induction (Fig. S5A) actually results from the addition of two independent factors of open complex destabilization: RNAP binding by ppGpp and DNA relaxation. Interestingly, the transcriptional response to ppGpp induction was also monitored in mutant cells where it is unable to bind RNAP, inhibiting its direct regulatory activity (52). Remarkably, almost half as many genes respond as in the wild-type cells (representing 35% of σ^70^ promoters, although with weaker magnitudes and slightly slower response times), and these promoters exhibit a similar (albeit weaker) sequence signature at the same location (Fig. S5B). A plausible explanation is that ppGpp induction indeed triggered DNA relaxation (29), resulting in a similar but partial response compared to that of wild-type cells. This scenario remains hypothetical, as the SC levels were not directly measured in these samples; it would likely involve a posttranscriptional effect of ppGpp on gyrase activity, as frequently occurs in response to stress or metabolic signals (9). This analysis also suggests a specific effect of ppGpp for the activation of promoters with A/T%-rich discriminators (54) (compare the non and activated curves in Fig. S5); this observation might be linked to the weak difference between these two groups in several data sets involving DNA relaxation (e.g., Fig. 4A and C), although the opposite is seen in other cases (e.g., Fig. 4B and 5A and C).

Altogether, this combined analysis of transcriptomic data fully confirms the notion that the regulation by SC relaxation and ppGpp is partially redundant in their transcriptional effect but distinct; as an example, the SC dependence of *hisR* was found to be independent of *relA* in S. enterica (55). More precisely, SC relaxation may be considered a more fundamental form of regulation relying on the basic infrastructure of transcription, whereas ppGpp synthesis may itself trigger DNA relaxation (but not conversely). The relationship between the two pathways is further emphasized by the observation that, in the evolution experiment, the two genes most quickly and robustly affected by mutations are *topA* and *spoT* (49, 56), involved precisely in SC and ppGpp synthesis/degradation (6), respectively. Interestingly, the *spoT* mutation alone explains only a part of the observed transcriptional change (57), while similarly, the *topA* mutation alone generates only a fraction of the observed signal at the discriminator (data not shown), suggesting a synergistic action of these two mutations (49, 56). The additive selection of promoters based on the same sequence signal at the discriminator provides a plausible and natural mechanistic explanation for this feature.

Finally, in the data sets obtained with environmental stress conditions that we have analyzed (Fig. 5), the genes associated with ppGpp synthesis are partly responsive but rather in an opposite direction to the discriminator sequence signature observed (repression in heat and oxidative stress, slight activation in cold stress), and this pathway does probably not contribute significantly to the observed signal.

## MATERIALS AND METHODS

### Synthetic promoters.

Sequences 230, 329, and 313 nt upstream of the *pheP*, *pelE*, and *pelD* start codons, respectively, were synthesized with mutations in the discriminator (GeneCust) and individually cloned into pUCTer-*luc* plasmids (see Table S1 in the supplemental material) upstream of a luciferase reporter gene (*luc*). E. coli strain MG1655 cells were then transformed with these plasmids using a standard electroporation procedure.

### Measurement of DNA relaxation response of mutant promoters *in vivo*.

E. coli cells carrying the plasmids with the different promoters were recovered from glycerol stock (−80°C) and grown overnight (about 16 h) on LB agar plates at 37°C. The obtained colonies were further transferred to liquid cultures overnight (about 16 h), with shaking at 200 rpm under selective antibiotic pressure (ampicillin at 60 μg/ml final concentration). LB medium was used for bacteria carrying plasmids with *pheP*-derived promoters, whereas M63 minimal medium supplemented with 0.2% glucose was used for bacteria carrying plasmids with *pelE-* and *pelD*-derived promoters. Cells were washed (2× centrifugation at 8,000 rpm), and then outgrowth cultures were performed in the same medium without antibiotics, stopped during exponential phase, and diluted for a final optical density at 600 nm (OD_600_) of 0.1 in a 96-well microplate. Each well (200 μl final volume) contained the chosen medium supplemented with d-luciferin (450 μg/ml final). The microplate was placed in a humidity cassette and grown at 37°C until stationary phase was reached in a microplate reader (Tecan Spark). The OD_600_ and luminescence were measured every 5 min, preceded by a 45-s shaking step (double orbital, 3.5-mm amplitude). During mid-exponential phase for *pheP* and early exponential phase for *pelE* and *pelD*, the microplate was taken out and DNA relaxation was transiently induced by injecting 5 μl of novobiocin (50, 100, 150, and 200 μg/ml final concentrations tested) using a multichannel pipette. Data files produced by the microplate reader were parsed using a Python home-made script, and the response to DNA relaxation was computed by comparing the luminescence values (in triplicates) of the novobiocin-shocked strain compared to the same strain injected with water (novobiocin solvent) 60 min after shock. The employed firefly luciferase has a short lifetime, between 6 min in B. subtilis (58) and 45 min in E. coli (59). Confidence intervals and *P* values were computed using Student statistics.

### Genome-wide analyses of discriminator sequences.

Transcriptomes obtained after DNA relaxation by antibiotics, inheritable supercoiling variations, or environmental stresses were collected from the literature, as were genome-wide TSS maps (Table S2). A scan for promoter motifs was conducted with bTSSfinder (60), imposing each TSS position at the experimentally determined nucleotide. Tables of detailed promoter sequences are provided in Table S3. For E. coli, the analysis was also tested with an alternate list of promoters (from the EcoCyc database [1]), which gave comparable results. In all expression data sets, genes were considered significantly activated/repressed under a common standard statistical selection procedure, based on a threshold of 0.05 on the adjusted *P* value, except for the evolution data (0.3; due to the otherwise low number of responsive genes; see details in reference 25). Promoters controlling several genes (operons) were considered differentially expressed if at least one gene of these genes is differentially expressed. For three data sets (heat and cold shock and *S. elongatus*), *P* values were not provided and were replaced by a threshold on log fold change values (±0.5), generating subsets of act/rep genes of sizes comparable to those in other data sets. For enterobacteria, only *σ*^70^-dependent promoters were retained and aligned at their −10 site to reduce statistical noise. Some of them also bind other *σ* factors, but *σ*^70^ is predominant in exponential phase where the analyzed samples were collected. The A/T% content was computed along 5-bp sliding windows (Fig. 3A and C). Promoters were classified according to their A/T% in a 5-nt window centered around position −2 rather than the entire discriminator (of variable size), which improves the statistical analysis while not affecting the distribution of promoters significantly (Fig. S3). For *S. elongatus* and M. pneumoniae, where the sigma factors differ from those of enterobacteria, all promoters were retained and aligned at their TSS. As expected due to the variable size of the discriminator, the resulting A/T% signal had a poorer signal definition (Fig. S4) and exhibited small positional shifts. For *S. elongatus*, the A/T% difference was observed slightly downstream of the TSS, and we used position +4 for the analysis. For M. pneumoniae, the A/T% peak was observed at position −6, and all positions were shifted by −4 nt to impose it at the −10 position. The relation between A/T% content and expression response was quantified either by linear regression (Fig. 3) or by a $\chi^{2}$ test between activated and repressed promoters (Fig. 4 and 6). All error bars shown are 95% confidence intervals, except the colored areas of Fig. 3 (67% confidence intervals). In all figures, statistical significance is illustrated based on the *P* value (***, *P < *0.001; **, 0.001<*P*<0.01; *, 0.01<*P*<0.05). Curves of Fig. 3 (A/T% profiles of promoters, linear regression) are provided in Fig. S4 for the other data sets.

### Model of transcriptional regulation by SC.

The observed correlation between promoter opening thermodynamics and expression strength (Fig. 1) is accounted for by a thermodynamic regulatory model (61):

$$k(\sigma ,s)=k_{0}\exp\left(\min\left(\frac{\Delta G(\sigma ,s)}{k_{B}T},0\right)\right)$$

where *k* is the transcription rate, *k*_0_ is the basal (maximal) rate, *s* is the precise 14-nt sequence of the denatured region in the open complex (62), and $k_{B}T$ is the Boltzmann factor. The free energy, ΔG, is composed of two contributions, the opening penalty, $\Delta G_{\text{op}}(\sigma ,s)$ (Fig. 1B), and an additional contribution representing the opening assistance by RNAP, $\Delta G_{P}^{0}(s)$:

$$\Delta G(\sigma ,s)=\Delta G_{\text{op}}(\sigma ,s)\;+\;\Delta G_{P}^{0}(s)$$

The opening energy, $\Delta G_{\text{op}}$, is computed from an established coarse-grained unidimensional description of DNA twist-dependent thermodynamics (63), where the total SC level is assumed to contribute to DNA opening by RNAP (neglecting any effect of its partitioning into twist/writhe and constrained/unconstrained contributions in the thermodynamic equilibrium of open complex formation). We hypothesize that $\Delta G_{P}^{0}(s)$ depends on the discriminator sequence, in agreement with direct measurements (32) and with the observation that the TSS position can be shifted by mutations in the discriminator (Fig. 1A) but is not affected by SC variations. At high negative SC levels, the opening penalty becomes negligible [$\Delta G_{\mathrm{op}}(\sigma ,s)+\Delta G_{P}^{0}(s)>0$] (Fig. 1B) and the maximal rate, *k*_0_, is achieved, whereas the promoter is mostly closed when DNA is strongly relaxed.

Based on these hypotheses, the expression fold change of a promoter during an SC variation (in the regimen where it is not fully activated) depends only on and is independent of the precise (usually unknown) value of $\Delta G_{P}^{0}(s)$:

$$\Delta G(\sigma_{0}\;+\;\Delta \sigma ,s)\;-\;\Delta G(\sigma_{0},s)\;=\;\Delta G_{\text{op}}(\sigma_{0}\;+\;\Delta \sigma ,s)\;-\;\Delta G_{\text{op}}(\sigma_{0},s)$$

For the modeling of the data in Fig. 1, where absolute levels of expression (and not just fold changes) were measured, we used a single fitted value, $\Delta G_{P}^{0}=3.5\;k_{B}T≃2$ kcal/mol, to avoid overparameterization. This approximation may explain the slight discrepancies with the data (Fig. 1F and G), but the overall agreement suggests that the sequence-dependent variations of $\Delta G_{P}^{0}(s)$ remain limited in the framework of our analysis. All following computations (for all promoters and species) were carried out with the same value of $\Delta G_{P}^{0}$, but since they involve expression fold changes (rather than absolute levels), the value of $\Delta G_{P}^{0}(s)$ for each promoter has a marginal effect on the predictions.

For each promoter, the denaturation energy is computed with TwistDNA (63) using the 14-bp sequence starting from (and including) the −10 hexamer, corresponding to the extent of the transcription bubble (flanked by 100-bp-long G-tracts to avoid boundary effects in the computation). The only adjustable parameter of TwistDNA is an effective salt concentration, which is calibrated on the data of Fig. 1 (21), yielding values of 1.5 mM and 3 mM for *in vitro* and *in vivo* transcription, respectively, the latter value being kept constant for all subsequent *in vivo* calculations. These low values are likely due to the strongly simplified description of the solvent (continuous distribution of monovalent ions) and DNA (unidimensional molecule) used in that software and should be considered effective parameters for the computation rather than quantitative concentrations.

Under all aforementioned approximations, it is possible to predict the quantitative regulatory effect of SC variations from their experimentally available genome-averaged value (e.g., using chloroquine-agarose gels). The validity of the computation is justified *a posteriori* by the good agreement with *in vitro* and *in vivo* expression data (Fig. 1 and 2). Note that, at the genomic scale, the SC level locally available to RNAP for the opening of a given promoter may deviate from the genome-averaged SC level because of many complicating factors beyond the simple model considered here (three-dimensional conformation of the promoter, binding of regulatory proteins and nucleoid-associated proteins, structural transitions occurring at nearby sites, etc.; see Discussion). However, because of the monotonous nature of the activation curves (Fig. 1B and E), all main results are robust when the SC levels are globally shifted by up to ±0.01.

### Superhelical densities.

*In vivo* SC levels used in the computations of Fig. 1 were taken from references 17, 21 (E. coli strains with norfloxacin), and 33 (topoisomerase mutants of E. coli).

Expression fold changes in response to relaxation measured in microplates with *pheP-*, *pelE-*, and *pelD*-derived promoters were reproduced (Fig. 2) with a relaxation magnitude, Δσ=0.001, starting from a level of σ=−0.032 in LB rich medium and σ=−0.023 for M63+G minimal medium. This low magnitude may be partly due to the slow growth conditions in microplates but mostly to a buffering effect of the reporter system (luciferase lifetime of several to tens of minutes) and should be considered an effective value used in the modeling, as also suggested by the low repressive effect of novobiocin compared to batch cultures (34).

For the computation of the genome-wide contribution to the relaxation response (see Discussion), transcription rates from all promoters are normalized by their sum under each condition before computing fold changes, without any cutoff value (consistent with transcriptomic analysis protocols). This procedure results in the activation of a fraction of promoters (since the G/C-rich promoters represent a weaker proportion of total transcripts after the relaxation, A/T-rich promoters appear activated; see Fig. S6). Levels of SC variations associated with all investigated conditions were reviewed in the literature (Table S2), exhibiting magnitudes in the range 0.01 to 0.015, with differences due to protocols in stress/shock conditions and chloroquine-agarose gel assays. To reduce the number of adjustable parameters (considering the heterogeneity of these data), all model predictions were computed with a single initial SC level, σ=−0.045 (a realistic value yielding the best overall agreement with observations), and a variation of Δσ=±0.015 (depending on the sign of the experimental response). The model predictions change only marginally when these figures are changed by less than 0.01 in either direction.

### Data availability.

See Table S2 for data availability information.

10.1128/mSystems.00978-21.1 TEXT S1 Insertions of *pelD-luc* and *pelE-luc* transcriptional fusions in *D. dadantii* chromosome. Download Text S1, PDF file, 0.04 MB. Copyright © 2021 Forquet et al. 2021 Forquet et al. https://creativecommons.org/licenses/by/4.0/ This content is distributed under the terms of the Creative Commons Attribution 4.0 International license.
