# Supplementary material for: Role of the Discriminator Sequence in the Supercoiling Sensitivity of Bacterial Promoters
Source: mSystems. 2021 Aug 24;6(4):e00978-21. doi: 10.1128/mSystems.00978-21 (PMC8422995; doi:10.1128/mSystems.00978-21)
Supplement: TABLE S1 [file msystems.00978-21-st001.pdf]

Promoter	Sequence
>pheP_WT_GCrich	CTCGAGTCAGAGGTGATGAGCCGGATTGCCGCGCCGATGATTGGCGGCATGATCACCGCACCTTTGCTGTCGCTGTTTATT<br>ATCCCGGCGGCGTATAAGCTGATGTGGCTGCACCGACATCGGGTACGGAAATAAAAGCAGGATACCCCGTTTAACCGTGTG<br>GATTGTGTCTTGCGACGATGGGCACTAAATGTTAAAAG**GTGCCC**CTCAACAAAAAAGACACACAGGGGAAAGGCGGATCC
>pheP_hybrid	CTCGAGTCAGAGGTGATGAGCCGGATTGCCGCGCCGATGATTGGCGGCATGATCACCGCACCTTTGCTGTCGCTGTTTATT<br>ATCCCGGCGGCGTATAAGCTGATGTGGCTGCACCGACATCGGGTACGGAAATAAAAGCAGGATACCCCGTTTAACCGTGTG<br>GATTGTGTCTTGCGACGATGGGCACTAAATGTTAAAAG**GTTACC**CTCAACAAAAAAGACACACAGGGGAAAGGCGGATCC
>pheP_ATrich	CTCGAGTCAGAGGTGATGAGCCGGATTGCCGCGCCGATGATTGGCGGCATGATCACCGCACCTTTGCTGTCGCTGTTTATT<br>ATCCCGGCGGCGTATAAGCTGATGTGGCTGCACCGACATCGGGTACGGAAATAAAAGCAGGATACCCCGTTTAACCGTGTG<br>GATTGTGTCTTGCGACGATGGGCACTAAATGTTAAAAG**GTTAAT**CTCAACAAAAAAGACACACAGGGGAAAGGCGGATCC
>pelE_WT_ATrich	CTCGAGTCGAAATTAAAAATATAAATAATTGATTTAAATCATAAAAAATAAAAAACCAAGTAACACAAAGTTACAAATACA<br>GTCAATAGTTTATTTTTTATTAATAAAAACCATTGTCATCATCGTGACAAAGTTCACAAAATAGACACTCAAACCGCATAA<br>ACATTCGCCAAAGCAAAAGATGAAATGGTATTTC**GTTTT**TAGACACACATGTAACAAATGGACACCATTTGGATCGCTCAC<br>TGAGCACACAAGGAAAATTGCCATGAACAACTCACGTATGTCTTCCGTTTCAACACAGAAAACAACAGGACGTTCTGCCTT<br>GGGAACCAAAGGGATCC
>pelE_GCrich	CTCGAGTCGAAATTAAAAATATAAATAATTGATTTAAATCATAAAAAATAAAAAACCAAGTAACACAAAGTTACAAATACA<br>GTCAATAGTTTATTTTTTATTAATAAAAACCATTGTCATCATCGTGACAAAGTTCACAAAATAGACACTCAAACCGCATAA<br>ACATTCGCCAAAGCAAAAGATGAAATGGTATTTC**GCGCC**TAGACACACATGTAACAAATGGACACCATTTGGATCGCTCAC<br>TGAGCACACAAGGAAAATTGCCATGAACAACTCACGTATGTCTTCCGTTTCAACACAGAAAACAACAGGACGTTCTGCCTT<br>GGGAACCAAAGGGATCC
>pelD_WT_GCrich	CTCGAGAACTGTTTGGGTTATTTCAGATAAAAACGCTTATACATATAGCTGAATTTAAAAGAAAAATTAATTCAACATTCA<br>TAACTAAAAGTTACCGGTCACGATCACACTTTAGATAAAATTAATTAGCCTCATAAAAAAAACGAGATTTTGATCACAAAAT<br>AAACAATCGAAAACGCTTAAAAAT**CCGCC**TTGCCAAAGGACAAAATGGCGTTTCATTTTTTTCACAAACACTTTTCAGTC<br>AACAAAATTGGATTAGCGCAGATAGCGCAAGGAACCAGTCTATGAACAACACACGAGTGTCTTCCGTAGGTACCAAGGATCC
>pelD_ATrich	CTCGAGAACTGTTTGGGTTATTTCAGATAAAAACGCTTATACATATAGCTGAATTTAAAAGAAAAATTAATTCAACATTCA<br>TAACTAAAAGTTACCGGTCACGATCACACTTTAGATAAAATTAATTAGCCTCATAAAAAAAACGAGATTTTGATCACAAAAT<br>AAACAATCGAAAACGCTTAAAAAT**CTTTT**TTGCCAAAGGACAAAATGGCGTTTCATTTTTTTCACAAACACTTTTCAGTC<br>AACAAAATTGGATTAGCGCAGATAGCGCAAGGAACCAGTCTATGAACAACACACGAGTGTCTTCCGTAGGTACCAAGGATCC
	XhoI restriction site, -35 element, -10 element, **discriminator**, TSS, BglII restriction site

Plasmid	Description	Origin
pGEMT	High-copy-number vector containing a multiple cloning site within the alpha-peptide coding region of the enzyme beta-galactosidase.	Promega
pGEMT-*pelA*-BglII-*pelE*	pGEMT derivative containing both the 500-bp region with *pelA-BglII* and the 500-bp region with *pelE*.	This work
pUCTer-*luc*	High-copy-number vector (pUC18 derivative) containing a multiple cloning site upstream of the *luc* reporter gene, followed by a *rrnB* terminator and a *cat* gene conferring chloramphenicol resistance.	Laboratory collection
pUCTer-*pelD*-*luc*	pUCTer-*luc* derivative containing the *D. dadantii pelD* WT promoter sequence above cloned upstream of the *luc* reporter gene.	This work
pUCTer-*pelE*-*luc*	pUCTer-*luc* derivative containing the *D. dadantii pelE* WT promoter sequence above cloned upstream of the *luc* reporter gene.	This work
**Primer name**	**Sequence**	
pelA F1	CTCAGGATAAAGGTAAGCTGC	
pelA R1	AGATCTGATGACGGTGTGGGCTAGACG	
pelE F1	CGTCTAGCCCACACCGTCATCAGATCTCGCCCGACTCGTCCCCTTTC	
pelE R1	GGAAGCGACTGAGACCATCATG	
pUCTer C18	GGGAGATCTACGACGTTGTAAAACGACGG	
pUCTer 155	GGGAGATCTAAAAGGCCATCCGTCAGGATGGCCTTCTCCGGGTCGAATTTGCTTTCG	
	rrnBT2 terminator sequence, BglII restriction site	
