# Supplementary figures and images for: Role of the Discriminator Sequence in the Supercoiling Sensitivity of Bacterial Promoters
Source: mSystems. 2021 Aug 24;6(4):e00978-21. doi: 10.1128/mSystems.00978-21 (PMC8422995; doi:10.1128/mSystems.00978-21)

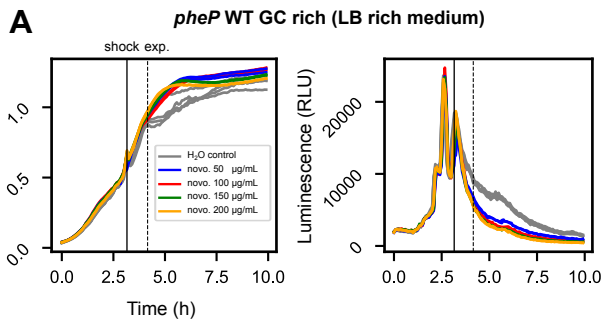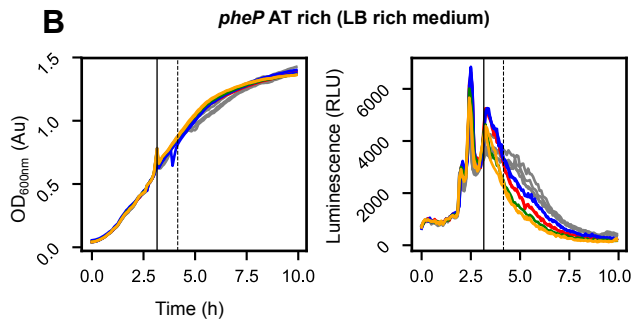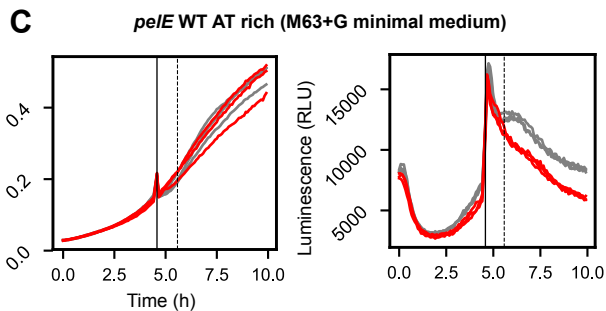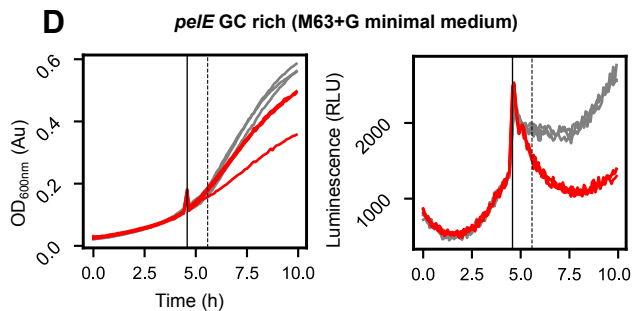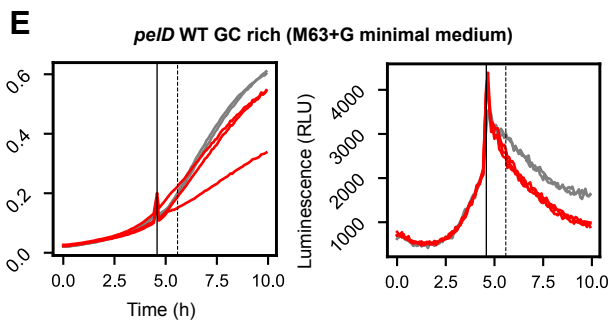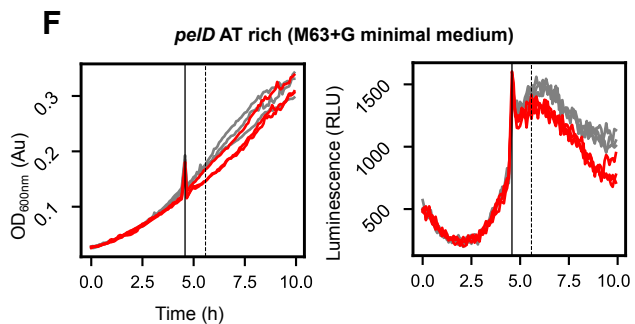

Supplement: FIG S2 [file msystems.00978-21-sf002.pdf]

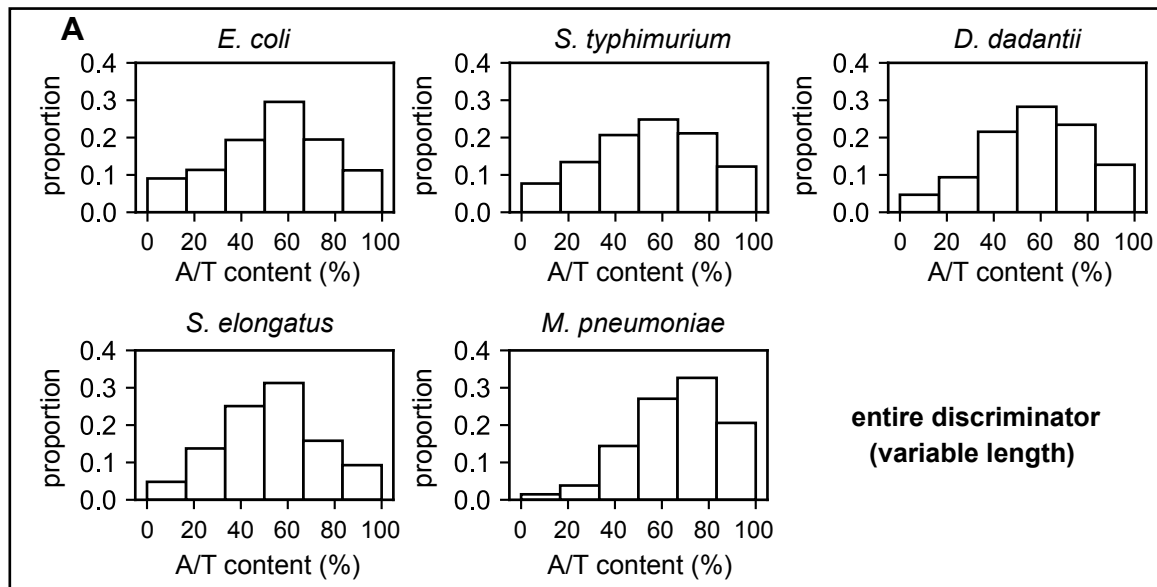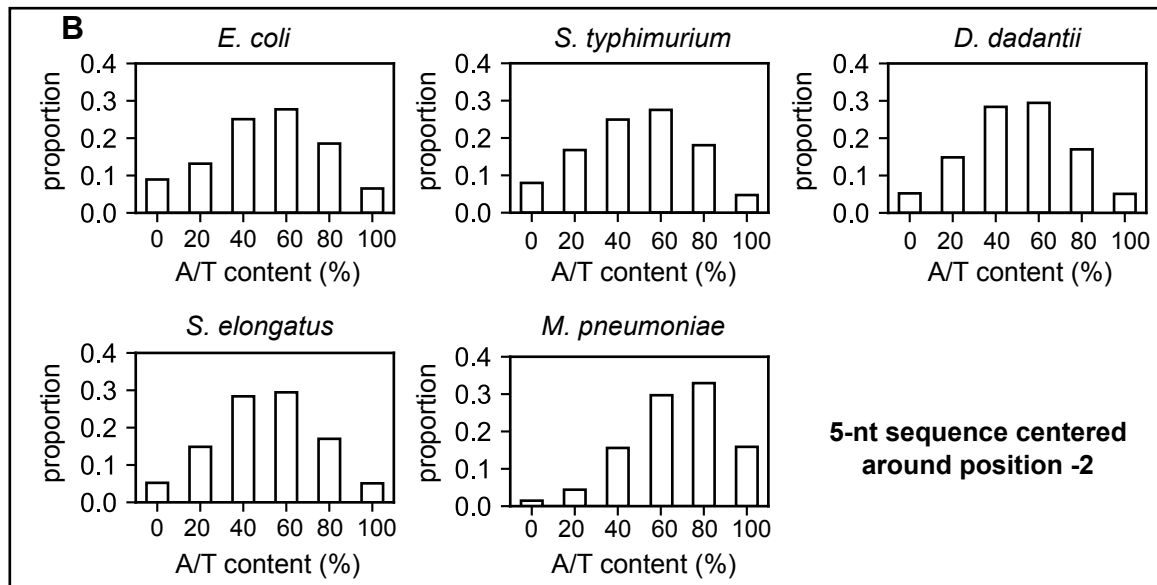

Supplement: FIG S3 [file msystems.00978-21-sf003.pdf]

**A** *E. coli*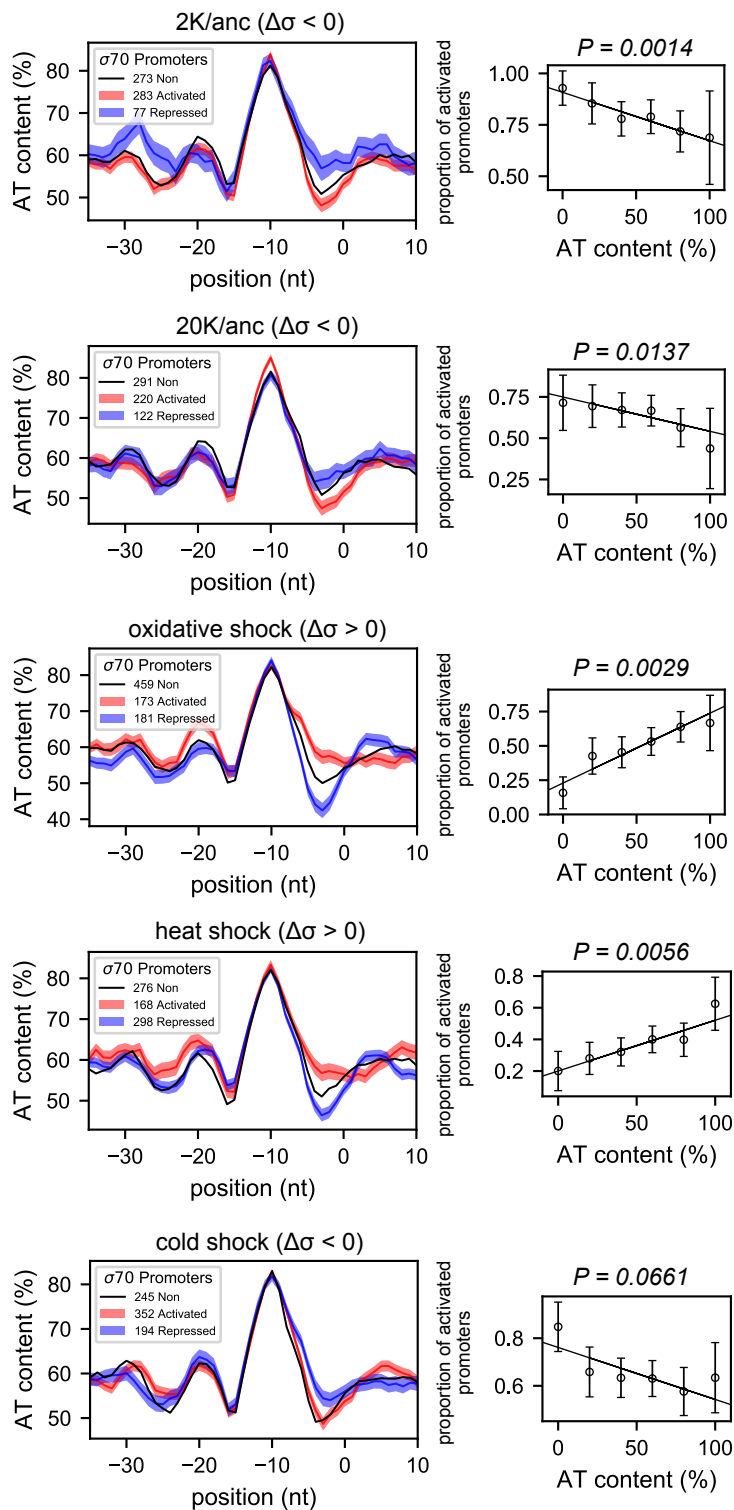**B** *D. dadantii*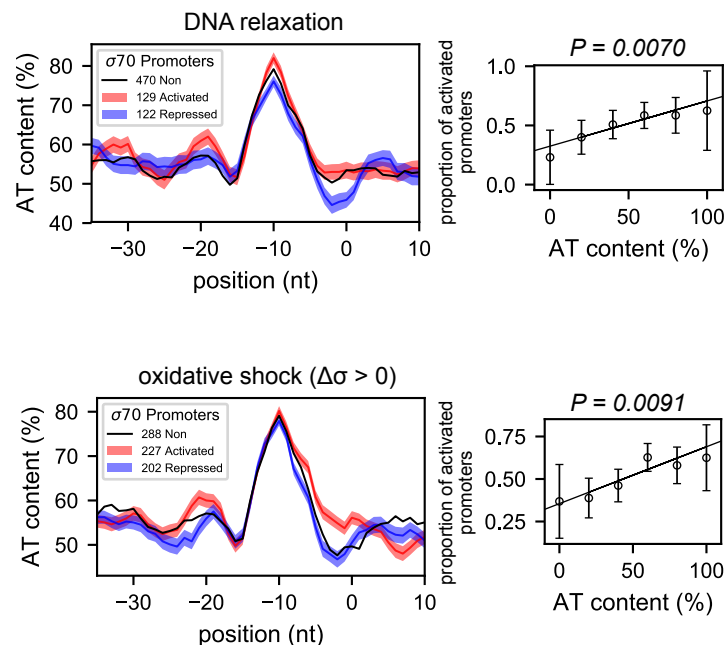**C** *S. elongatus*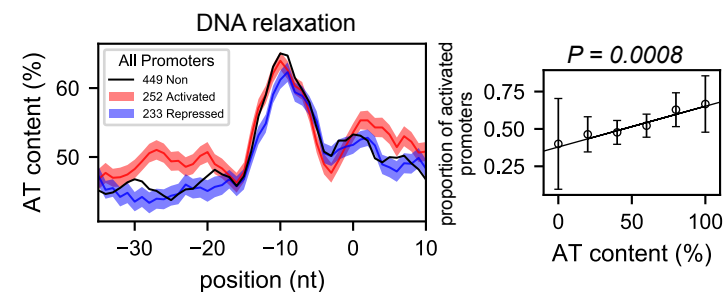**D** *M. pneumoniae*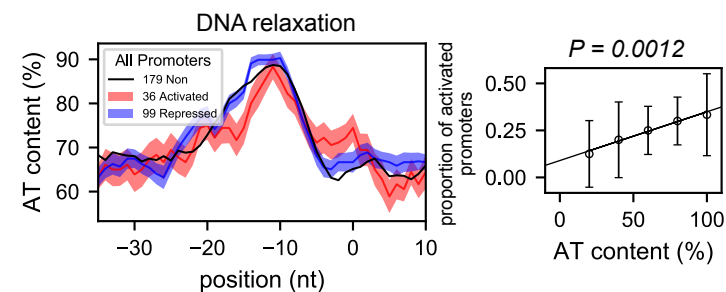

Supplement: FIG S4 [file msystems.00978-21-sf004.pdf]

proportion of  
activated promoters

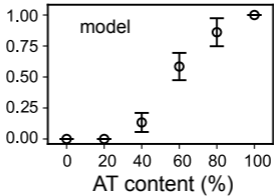

Supplement: FIG S6 [file msystems.00978-21-sf006.pdf]

**A** ppGpp binding (WT)

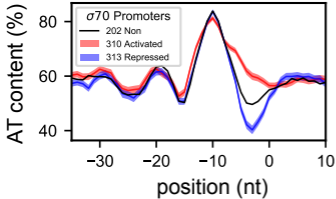

**B** no ppGpp binding

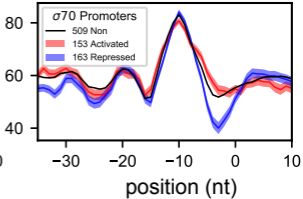

Supplement: FIG S5 [file msystems.00978-21-sf005.pdf]
