# Supplementary material for: Role of the Discriminator Sequence in the Supercoiling Sensitivity of Bacterial Promoters
Source: mSystems. 2021 Aug 24;6(4):e00978-21. doi: 10.1128/mSystems.00978-21 (PMC8422995; doi:10.1128/mSystems.00978-21)
Supplement: FIG S1 [file msystems.00978-21-sf001.pdf]

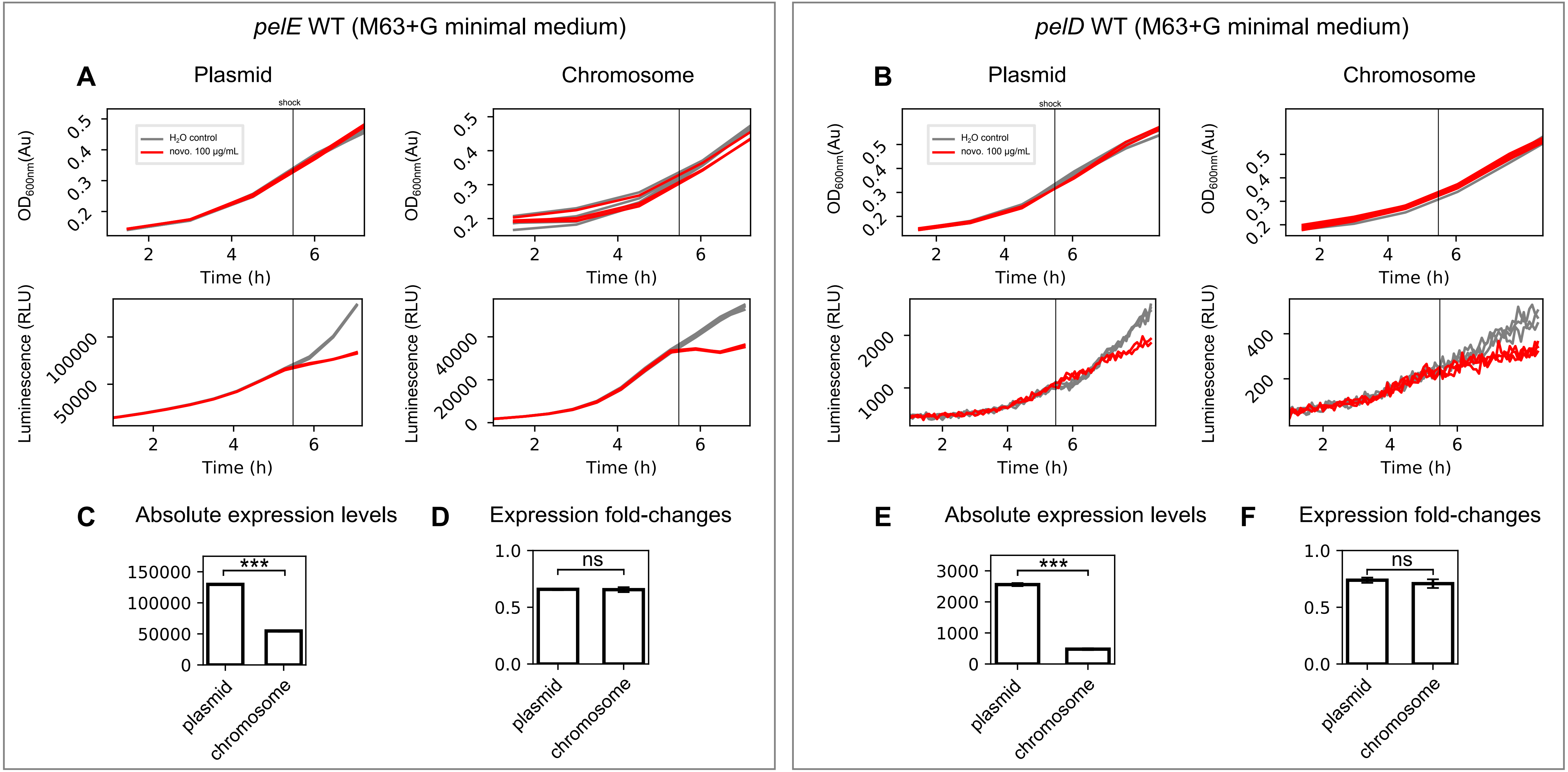

pelE WT (M63+G minimal medium)
A
Plasmid
Chromosome
shock
H2O control
novo. 100 µg/mL
OD600nm(Au)
Time (h)
Luminescence (RLU)
C
Absolute expression levels
***
plasmid
chromosome
D
Expression fold-changes
ns
pelD WT (M63+G minimal medium)
B
E
F
