# Supplementary material for: Role of the Discriminator Sequence in the Supercoiling Sensitivity of Bacterial Promoters
Source: mSystems. 2021 Aug 24;6(4):e00978-21. doi: 10.1128/mSystems.00978-21 (PMC8422995; doi:10.1128/mSystems.00978-21)
Supplement: TABLE S2 [file msystems.00978-21-st002.pdf]

**Species**	**Condition**	**Condition reference**	**SC variation**	**SC variation reference**	**Transcription start sites reference**	**A/T% difference p-value (act vs rep)**	**Model sensitivity gain (p-value)**
Salmonella typhimurium	novobiocin	[1]	-	no measurement	[2]	$< 10^{-5}$	13.8% ($< 10^{-6}$)
Dickeya dadantii	novobiocin	[3]	-	[4]	[5]	< 0.001	9.8% (< 0.001)
Escherichia coli	norfloxacin	[6]	-	[6]	[7]	0.010	5.1% (0.039)
Synechococcus elongatus	correlation	[8]	-	[8]	[9]	0.004	4.7% (0.099)
Mycoplasma pneumoniae	novobiocin	[10]	-	no measurement	[10]	0.029	7.8% (0.020)
Escherichia coli	heat shock	[11]	-	[12]	[7]	$< 10^{-5}$	7.5% ($< 10^{-4}$)
	cold shock	[13]	+	[14]	[7]	0.007	4.1% (0.038)
	oxidative shock	[15]	-	[16]	[7]	$< 10^{-7}$	9.0% (0.013)
Dickeya dadantii	oxidative shock	[17]	-	[4]	[5]	$< 10^{-4}$	7.6% (< 0.001)
Escherichia coli	experimental evolution (2K mutant)	[3]	+	[18]	[7]	0.005	3.7% ((0.011)
	experimental evolution (20K mutant)	[3]	+	[18]	[7]	0.011	1.8% (0.18)

References

[1] Gogoleva, N. E., Konnova, T. A., Balkin, A. S., Plotnikov, A. O., and Gogolev, Y. V. (2020) Transcriptomic data of Salmonella enterica subsp. enterica serovar Typhimurium str. 14028S treated with novobiocin. *Data in Brief,* **29**.

[2] Kröger, C., Dillon, S. C., Cameron, A. D. S., Papenfort, K., Sivasankaran, S. K., Hokamp, K., Chao, Y., Sittka, A., Hébrard, M., Händler, K., Colgan, A., Leekitcharoenphon, P., Langridge, G. C., Lohan, A. J., Loftus, B., Lucchini, S., Ussery, D. W., Dorman, C. J., Thomson, N. R., Vogel, J., and Hinton, J. C. D. (2012) The transcriptional landscape and small RNAs of Salmonella enterica serovar Typhimurium. *Proceedings of the National Academy of Sciences of the United States of America,* **109**(20), 1277–1286.

[3] El Houdaigui, B., Forquet, R., Hindré, T., Schneider, D., Nasser, W., Reverchon, S., and Meyer, S. (2019) Bacterial genome architecture shapes global transcriptional regulation by DNA supercoiling. *Nucleic Acids Research,* **47**(11), 5648–5657.

[4] Ouafa, Z.-A., Reverchon, S., Lautier, T., Muskhelishvili, G., and Nasser, W. (2012) The nucleoid-associated proteins H-NS and FIS modulate the DNA supercoiling response of the pel genes, the major virulence factors in the plant pathogen bacterium Dickeya dadantii. *Nucleic Acids Research,* **40**(10), 4306–4319.

[5] Forquet, R., Jiang, X., Nasser, W., Hommais, F., Reverchon, S., and Meyer, S. (2020) Mapping the complex transcriptional landscape of the phytopathogenic bacterium Dickeya dadantii. *bioRxiv,* p. 2020.09.30.320440.

[6] Blot, N., Mavathur, R., Geertz, M., Travers, A., and Muskhelishvili, G. (2006) Homeostatic regulation of supercoiling sensitivity coordinates transcription of the bacterial genome. *EMBO Reports,* **7**(7), 710–715.

[7] Conway, T., Creecy, J. P., Maddox, S. M., Grissom, J. E., Conkle, T. L., Shadid, T. M., Teramoto, J., San Miguel, P., Shimada, T., Ishihama, A., Mori, H., and Wanner, B. L. (2014) Unprecedented high-resolution view of bacterial operon architecture revealed by RNA sequencing. *mBio,* **5**(4), 01442–01414.

[8] Vijayan, V., Zuzow, R., and O'Shea, E. K. (2009) Oscillations in supercoiling drive circadian gene expression in cyanobacteria. *Proceedings of the National Academy of Sciences of the United States of America,* **106**(52), 22564–22568.

[9] Vijayan, V., Jain, I. H., and O'Shea, E. K. (2011) A high resolution map of a cyanobacterial transcriptome. *Genome Biology,* **12**(5), 47.

[10] Junier, I., Unal, E. B., Yus, E., Lloréns-Rico, V., and Serrano, L. (2016) Insights into the Mechanisms of Basal Coordination of Transcription Using a Genome-Reduced Bacterium. *Cell Systems,* **2**(6), 391–401.

[11] Bartholomäus, A., Fedyunin, I., Feist, P., Sin, C., Zhang, G., Valleriani, A., and Ignatova, Z. (2016) Bacteria differently regulate mRNA abundance to specifically respond to various stresses. *Philosophical Transactions. Series A, Mathematical, Physical, and Engineering Sciences,* **374**(2063).

[12] Ogata, Y., Mizushima, T., Kataoka, K., Miki, T., and Sekimizu, K. (1994) Identification of DNA topoisomerases involved in immediate and transient DNA relaxation induced by heat shock in Escherichia coli. *Molecular & general genetics: MGG,* **244**(5), 451–455.

[13] Zhang, Y., Burkhardt, D. H., Rouskin, S., Li, G.-W., Weissman, J. S., and Gross, C. A. (April, 2018) A Stress Response that Monitors and Regulates mRNA Structure Is Central to Cold Shock Adaptation. *Molecular Cell,* **70**(2), 274–286.e7.

[14] Mizushima, T., Kataoka, K., Ogata, Y., Inoue, R.-i., and Sekimizu, K. (1997) Increase in negative supercoiling of plasmid DNA in Escherichia coli exposed to cold shock. *Molecular Microbiology,* **23**(2), 381–386.

[15] Jozefczuk, S., Klie, S., Catchpole, G., Szymanski, J., Cuadros-Inostroza, A., Steinhauser, D., Selbig, J., and Willmitzer, L. (2010) Metabolomic and transcriptomic stress response of Escherichia coli. *Molecular Systems Biology,* **6**, 364.

[16] Weinstein-Fischer, D., Elgrably-weiss, M., and Altuvia, S. (2000) Escherichia coli response to hydrogen peroxide: a role for DNA supercoiling, Topoisomerase I and Fis. *Molecular Microbiology,* **35**(6), 1413–1420.

[17] Jiang, X., Sobetzko, P., Nasser, W., Reverchon, S., and Muskhelishvili, G. (2015) Chromosomal "Stress-Response" Domains Govern the Spatiotemporal Expression of the Bacterial Virulence Program. *mBio,* **6**(3), e00353–15.

[18] Crozat, E., Philippe, N., Lenski, R. E., Geiselmann, J., and Schneider, D. (2005) Long-term experimental evolution in Escherichia coli. XII. DNA topology as a key target of selection. *Genetics,* **169**(2), 523–532.
