# Supplementary material for: Role of the Discriminator Sequence in the Supercoiling Sensitivity of Bacterial Promoters
Source: mSystems. 2021 Aug 24;6(4):e00978-21. doi: 10.1128/mSystems.00978-21 (PMC8422995; doi:10.1128/mSystems.00978-21)
Supplement: TEXT S1 [file msystems.00978-21-s0001.pdf]

Supplementary text

Insertions of *pelD-luc* and *pelE-luc* transcriptional fusions in *D. dadantii* chromosome

All Plasmids and primers used for these constructions are described in Supplementary Tab. S1. *D. dadantii* 3937-derivative strains A5740 and A5720 with *pelD-luc* and *pelE-luc* transcriptional fusions, respectively, inserted in the *pelA-pelE* intergenic region of the chromosome, were obtained as follow. First, two PCR products of 500 bp, corresponding to the *pelA* and *pelE* genes flanking the site of insertion, were obtained using primer pairs *pelAF1/pelAR1* and *pelEF1/pelER1*. Primers *pelAR1* and *pelEF1* included a unique restriction site for BglII and were designed to have a 20-bp short overlapping complementary sequence. The two resulting PCR products were fused by overlapping PCR using primers *pelAF1* and *pelER1*. The resulting *pelA-BglII-pelE* PCR product was cloned into pGEMT plasmid (pGEMT-*pelA*-BglII-*pelE*). Then, PCR fragments containing either *pelD-luc-CmR* or *pelE-luc-CmR* were obtained from plasmids pUCTer-*pelD-luc* and pUCTer-*pelE-luc*, respectively, by using primer pairs C18 and 155 with a BglII restriction site at their 5' extremities. Finally, the pGEMT-*pelA*-BglII-*pelE* plasmid and the PCR *pelD-luc-CmR* or *pelE-luc-CmR* fragments were digested with BglII and ligated. After transformation in *E. coli*, plasmids containing the expected insertions were selected and electroporated into *D. dadantii* strain 3937 using a standard electroporation procedure. The two insertions were introduced into *D. dadantii* chromosome by marker exchange recombination between chromosomal and plasmid-borne alleles. The recombinants were selected after successive cultures in low phosphate medium in the presence of chloramphenicol, conditions in which pGEMT derivatives are very unstable. The recombination was finally validated by PCR. *D. dadantii* strain 3937 cells were also transformed with plasmids carrying *pelE* and *pelD* native promoters (pUCTer-*pelE-luc* and pUCTer-*pelD-luc*, respectively, Supplementary Tab. S1) for further comparison with chromosomal fusions.
